# Supplementary material for: Reconstitution defines the roles of p62, NBR1 and TAX1BP1 in ubiquitin condensate formation and autophagy initiation
Source: Nat Commun. 2021 Sep 1;12:5212. doi: 10.1038/s41467-021-25572-w (PMC8410870; doi:10.1038/s41467-021-25572-w)
Supplement: Supplementary file 1 — Supplementary information [file 41467_2021_25572_MOESM1_ESM.pdf]

Supplementary information

Reconstitution defines the roles of p62, NBR1 and TAX1BP1 in ubiquitin condensate formation and autophagy initiation

Eleonora Turco[1,2*], Adriana Savova[1,2], Flora Gere[1], Luca Ferrari[1], Julia Romanov[1], Martina Schuschnig[1] and Sascha Martens[1,*]

[1]Max Perutz Labs, University of Vienna, Vienna BioCenter (VBC), Dr. Bohr-Gasse 9, 1030 Vienna, Austria
[2]These authors contributed equally

* eleonora.turco@univie.ac.at, sascha.martens@univie.ac.at

Supplementary figure 1

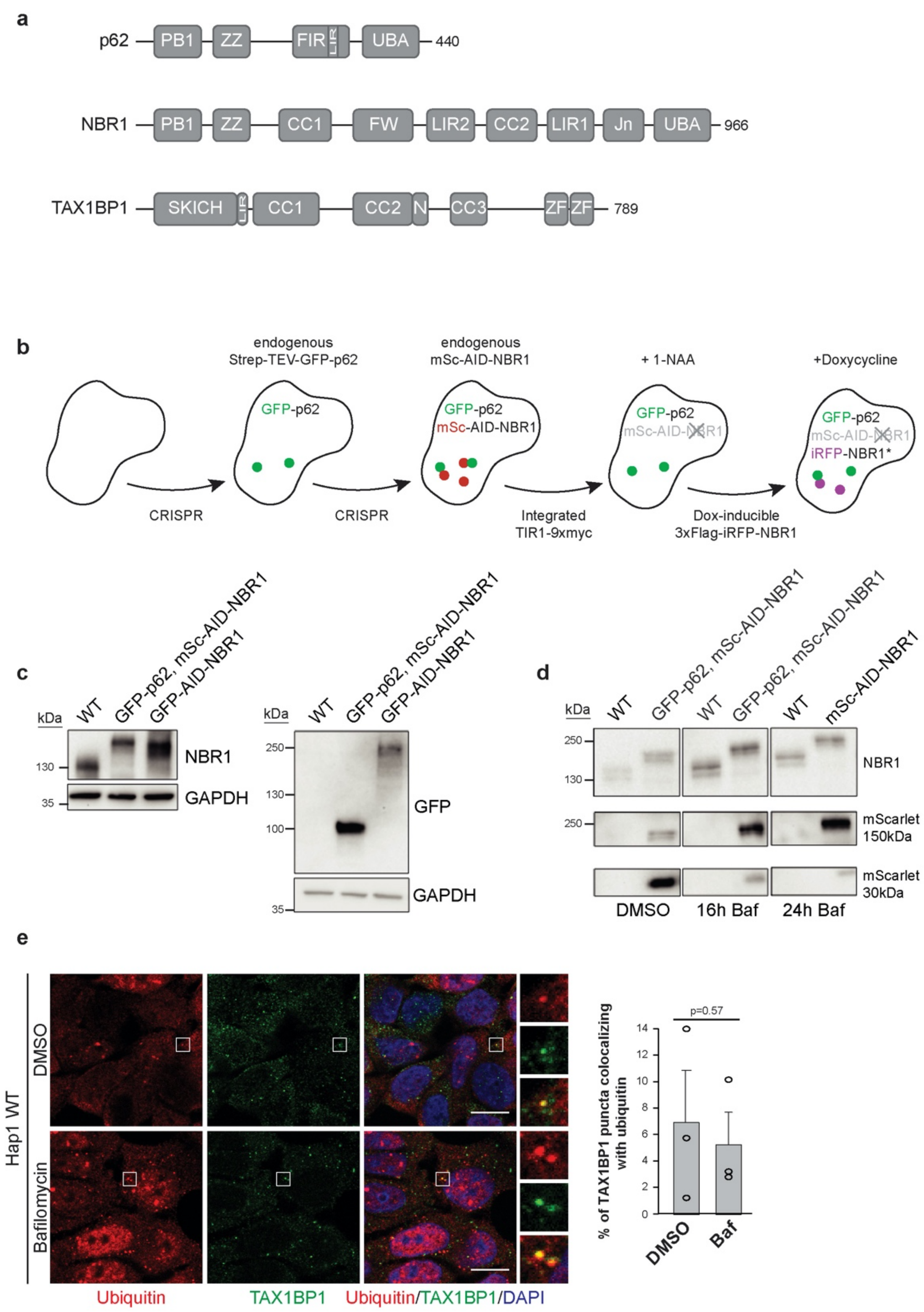

Supplementary figure 1

a) Schematic representation of the cargo receptors p62, NBR1 and TAX1BP1 and their domains.

b) Schematic representation of the CRISPR approach used to obtain the HAP1 GFP-p62, mSc-AID-NBR1, +/- TIR1, +/- iRFP-NBR1 cell lines used in Fig. 1a, Supplementary Fig. 1c, d, Fig. 3 and Supplementary Fig. 3. For details see the Method section.

c) Western blot validation of the endogenous protein tagging for the cell lines used in Fig. 1a, 3, Supplementary fig. 3 and Fig. 5d. HAP1 cells (WT or mSc-AID-NBR1, GFP-p62 or GFP-AID-NBR1) were lysed and analyzed by western blot. NBR1 antibody was used to detect the size shift in NBR1 caused by the addition of the tag. GFP antibody was used to detect the GFP tag fused to endogenous p62 or NBR1. For various experiments the proteins from the lysed cells consistently run at the molecular weights shown in the figure. Uncropped blots are provided as a Source Data file.

d) Validation of the GFP-p62, mSc-AID-NBR1 cell line used in Fig. 1a, 3a and Supplementary fig. 3b. HAP1 WT or GFP-p62, mSc-AID-NBR1 were left untreated (DMSO) or treated with bafilomycin (400 nM) for the indicated time. Cell lysates were analyzed by western blot. Proper tagging of endogenous NBR1 was assessed with NBR1 and mScarlet antibodies. For various experiments the proteins from the lysed cells consistently run at the molecular weights shown in the figure. Uncropped blots are provided as a Source Data file.

e) Colocalization of TAX1BP1 with ubiquitin in HAP1 WT cells mock-treated with DMSO or treated with bafilomycin (400 nM) for 2 h. Ubiquitin and TAX1BP1 were detected by immunofluorescence staining. Scale bar, 10 µm. For the colocalization analysis, average percentages of colocalization ± SEM for n = 3 are plotted. An unpaired, two-tailed Student's t test was used to estimate significance. P values are indicated in the figure.

Supplementary figure 2

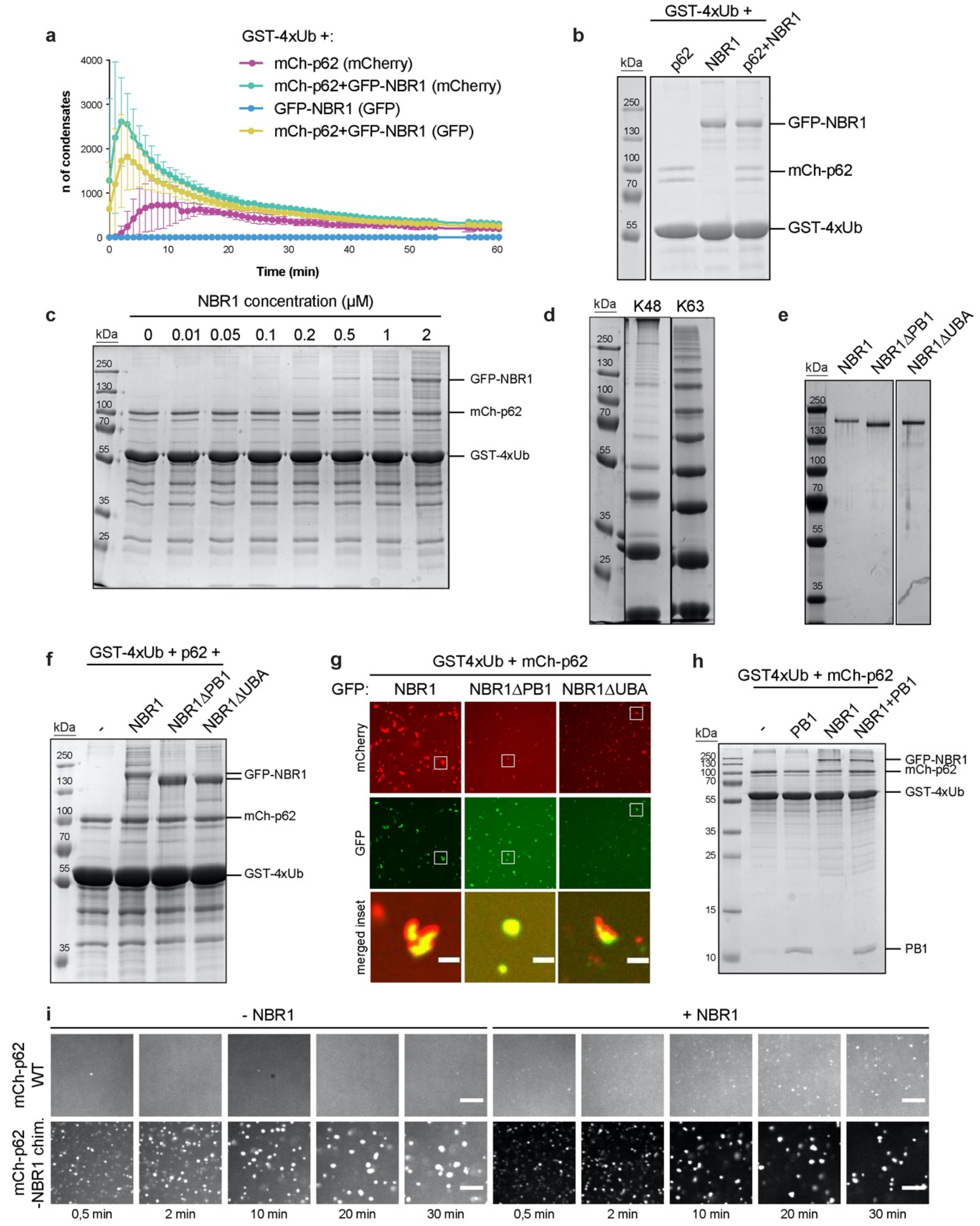

Supplementary figure 2.

a) Quantification of the condensate formation assay in Fig. 2b (GFP signal) and the relative control experiments with GST-4xUb, mCh-p62 (2 µM), GFP-NBR1 (2 µM) or a combination of them. The average number of condensates in the GFP or mCherry channels and standard deviations for n = 3 are plotted against time. SDS-Page gel with protein inputs for the

experiments is shown in Supplementary fig. 2b. Source data are provided as a Source Data file.

b, c) Condensate formation reactions for the experiments in Fig. 2b, c and Supplementary fig. 2a (**b**) and Fig. 2d (**c**) were recovered from the microscopy plate after imaging and analyzed by SDS-Page followed by Coomassie staining. n=3

d) Coomassie stained SDS-Page gel showing *in vitro* synthesized K48- and K63- linked ubiquitin chains (40 µg total ubiquitin/gel lane) used in Fig. 2e. n=3

e) Silver stained SDS-Page gel showing purified recombinant GFP-NBR1 WT, ΔPB1 and ΔUBA (1 µg each) used for the assays in Fig. 2f, g and h. n=1

f) Condensate formation reactions for the experiment in Fig. 2h were recovered from the microscopy plate and analyzed by SDS-Page gel followed by Coomassie staining. n=3

g) Condensate formation assay relative to the quantification in Fig. 2h. Representative images for the mCherry and GFP channels for the reactions containing the indicated proteins after 60 min incubation. Scale bar = 5µM, n=3.

h) Condensate formation reactions from Fig. 2i were recovered from the microscopy plate after imaging and analyzed by SDS-Page followed by Coomassie staining. n=3

i) Condensate formation assay with GST-4xUb (5 µM) and mCh-p62 WT (2µM) or the mCh-p62-NBR1 chimera (2µM), with or without NBR1 (1µM). Condensate formation over time in the mCherry channel was followed by spinning disk microscopy. Scale bar: 10 µm. n=3.

Uncropped gels for panels b, c, d, e, f, h are provided as a Source Data file.

Supplementary figure 3

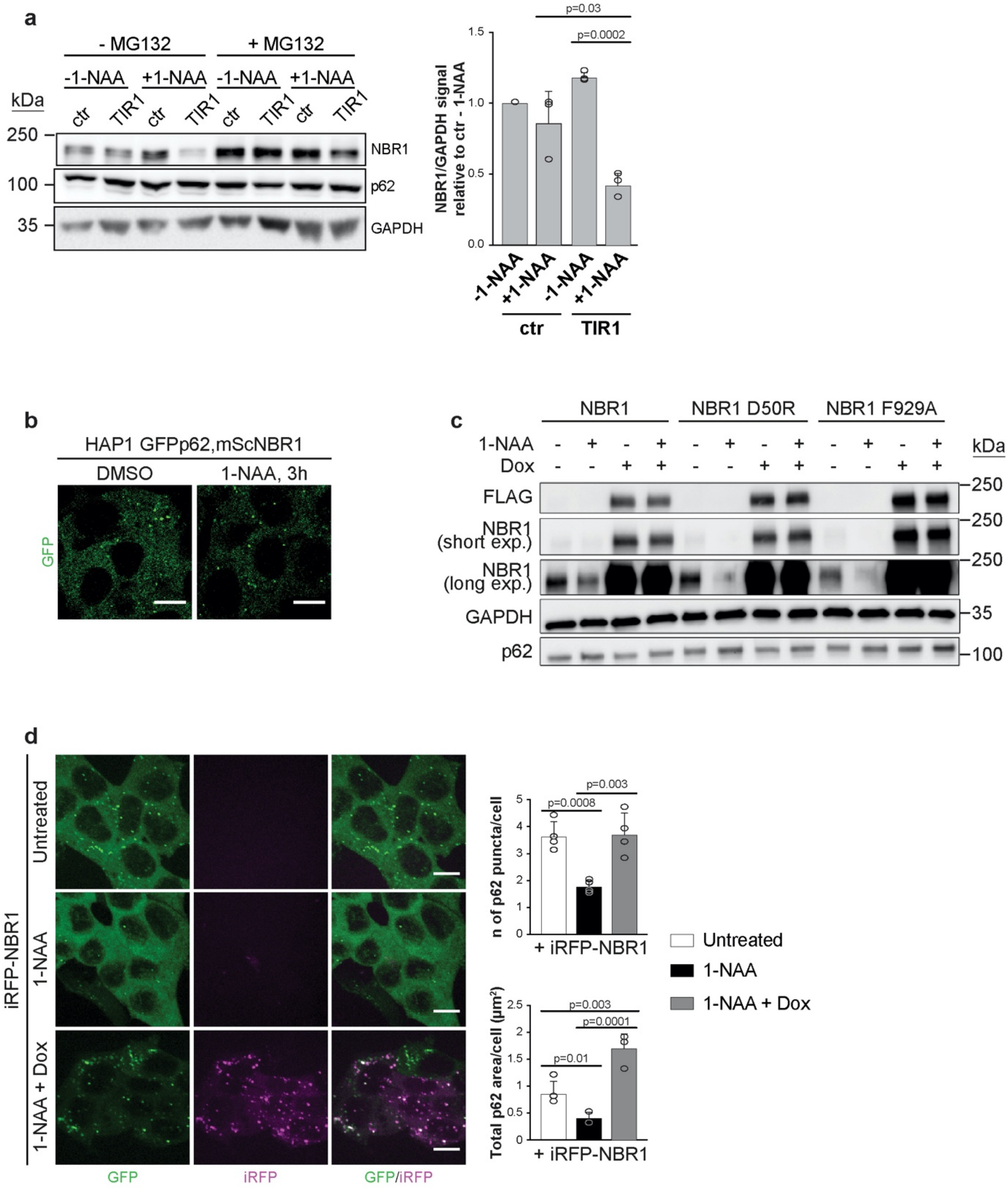

Supplementary figure 3.

a) Characterization of the HAP1 mSc-AID-NBR1, GFP-p62, TIR1 cell line. The indicated cell line or the control (ctr) cell line not expressing TIR1 were left untreated or treated with 500 µM 1-NAA, 10 µM MG132 or a combination of them for 3 h. Expression levels of NBR1 and p62 in the different conditions were evaluated by western blot. NBR1 bands intensity, for the samples not treated with MG132, was normalized to GAPDH intensity and plotted relative to

the untreated control cell line. Average band intensity and standard deviation for n = 3 are shown. Significant differences are indicated with * when p value ≤ 0.05, with ** when p value ≤ 0.01, with *** when p value ≤ 0.001. An unpaired, two-tailed Student's t test was used to estimate significance. P values are indicated in the figure. Uncropped blots are provided as a Source Data file.

b) Control experiment for Fig. 3b. The control HAP1, mSc-AID-NBR1, GFP-p62 cell line not expressing TIR1 was left untreated or treated with 1-NAA for 3 h. After treatment, GFP-p62 puncta formation was followed by live spinning disk microscopy. n=3.

c) Related to Fig. 3d, e and Supplementary fig. 3d. HAP1 mSc-AID-NBR1, GFP-p62, TIR1 cell lines, stably expressing doxycyclin inducible iRFP-NBR1 WT, D50R or F929A mutants were left untreated or treated with 1-NAA (1 mM for NBR1 WT rescue cells, 500 µM for the mutants), 50 ng/ml doxycyclin or a combination of them for 12 h. Cells lysates were analyzed by western blot. Expression levels of the depleted endogenous NBR1 and the doxycycline induced iRFP-NBR1 were assessed by western blot with anti-NBR1 and anti-FLAG antibodies respectively. Expression levels of p62 were also monitored in all conditions and GAPDH was used as loading control. n=3. Uncropped blots are provided as a Source Data file.

d) Related to Fig. 3d-f. HAP1 mSc-AID-NBR1, GFP-p62, TIR1 cells stably transfected with doxycyclin inducible FLAG-iRFP-NBR1 WT were left untreated or treated with 1-NAA, doxycyclin or a combination of them for 12 h. After treatment GFP-p62 puncta formation was followed by live cell imaging. Scale bar, 10 µm. The average number (top) and size (bottom) of p62 puncta and standard deviations for n = 4 are plotted. Significant differences are indicated with * when p value ≤ 0.05, with ** when p value ≤ 0.01, with *** when p value ≤ 0.001. An unpaired, two-tailed Student's t test was used to estimate significance. P values are indicated in the figure.

Supplementary figure 4

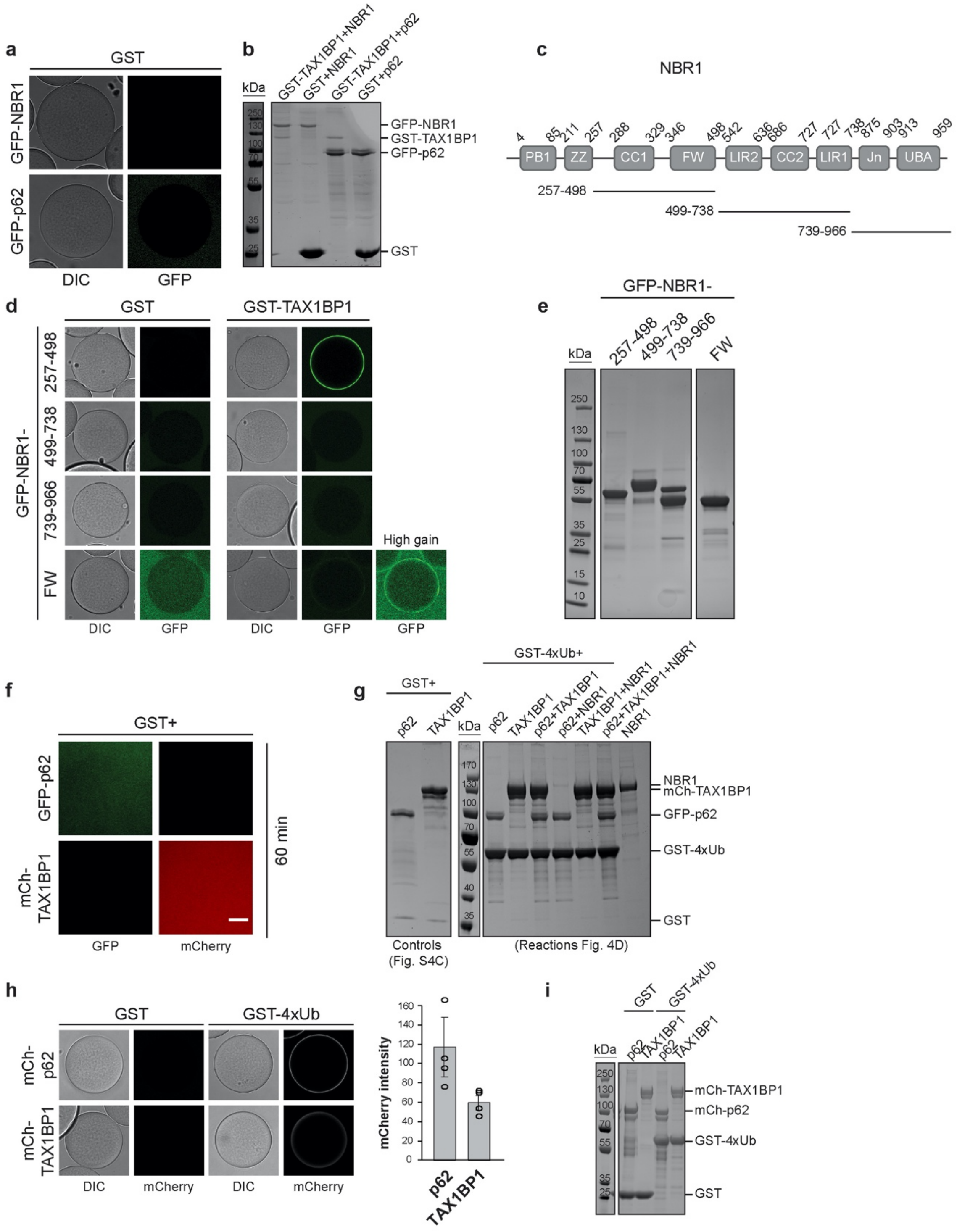

Supplementary figure 4.

a) Related to Fig. 4a. Glutathione beads were coupled with GST and incubated with GFP-p62 (2 µM) or GFP-NBR1 (2 µM). After 30 min incubation at room temperature beads were imaged at the equilibrium by confocal fluorescence microscopy. The experiment was done in three independent replicates. Protein inputs for this experiment are shown in Supplementary fig. 4b.

b) Pull-down reactions from Fig. 4a and Supplementary fig. 4a were recovered from the microscopy plate after imaging and analyzed by SDS-Page followed by Coomassie staining (n=3).

c) Schematic representation of NBR1 domains. The GFP-NBR1 constructs used in Supplementary fig. 4d, e are shown below the protein scheme. Abbreviations: PB1-Phox and Bem1 domain; ZZ-zinc finger; CC1-coiled coil 1; FW-four tryptophane domain; LIR2-LC3 interaction region area 2; CC2-coiled coil 2; LIR1-LC3 interaction region 1; Jn-juxta UBA domain; UBA-ubiquitin associated domain.

d) GST or GST-TAX1BP1 coated glutathione beads were incubated with the indicated GFP-tagged NBR1 fragments (2 µM). After 30 min incubation at RT beads were imaged at the equilibrium by LSM700 confocal microscope. All the samples were imaged using the same microscopy settings. For the GFP-NBR1-FW sample, the same beads were imaged also with higher gain for better visualization. The corresponding GST control for this sample was imaged with the same high gain settings (n=2). GFP-NBR1 protein inputs are shown in Supplementary fig. 4e.

e) 10 µM dilutions of the purified recombinant GFP-NBR1 constructs used for the pull-down experiment in Supplementary fig. 4d were visualized on Coomassie stained SDS-Page gel (n=2).

f) Negative controls for the condensate formation assay in Fig. 4d. GST (5 µM) was incubated with GFP-p62 (2 µM) or mCh-TAX1BP1 (2 µM) for 60 min and the reactions imaged by Spinning disk microscope. Scale bar: 10 µm. (n=3).

g) Condensate formation reactions from Fig. 4d and Supplementary fig. 4f were recovered from the microscopy plates after imaging and analyzed by SDS-Page followed by Coomassie staining (n=3). The purified recombinant NBR1 protein (5 µg) was additionally visualized in a separate lane of the gel (last lane).

h) GST or GST-4xUb coated glutathione beads were incubated with mCherry-p62 (2 µM) or mCherry-TAX1BP1 (2 µM) for 30 min at room temperature and imaged at the equilibrium by LSM700 confocal microscope. Representative beads are shown. The average mCherry signal on the beads and SEM for n = 3 are plotted. Protein inputs for the pull-down reactions are shown in Supplementary fig. 4i.

i) Samples from the pull-down experiment in Supplementary fig. 4h were collected from the microscopy plate after imaging and analyzed by SDS-Page followed by Coomassie staining (n=3).

Uncropped gels for panels b, e, g, I are provided as a Source Data file.

Supplementary figure 5

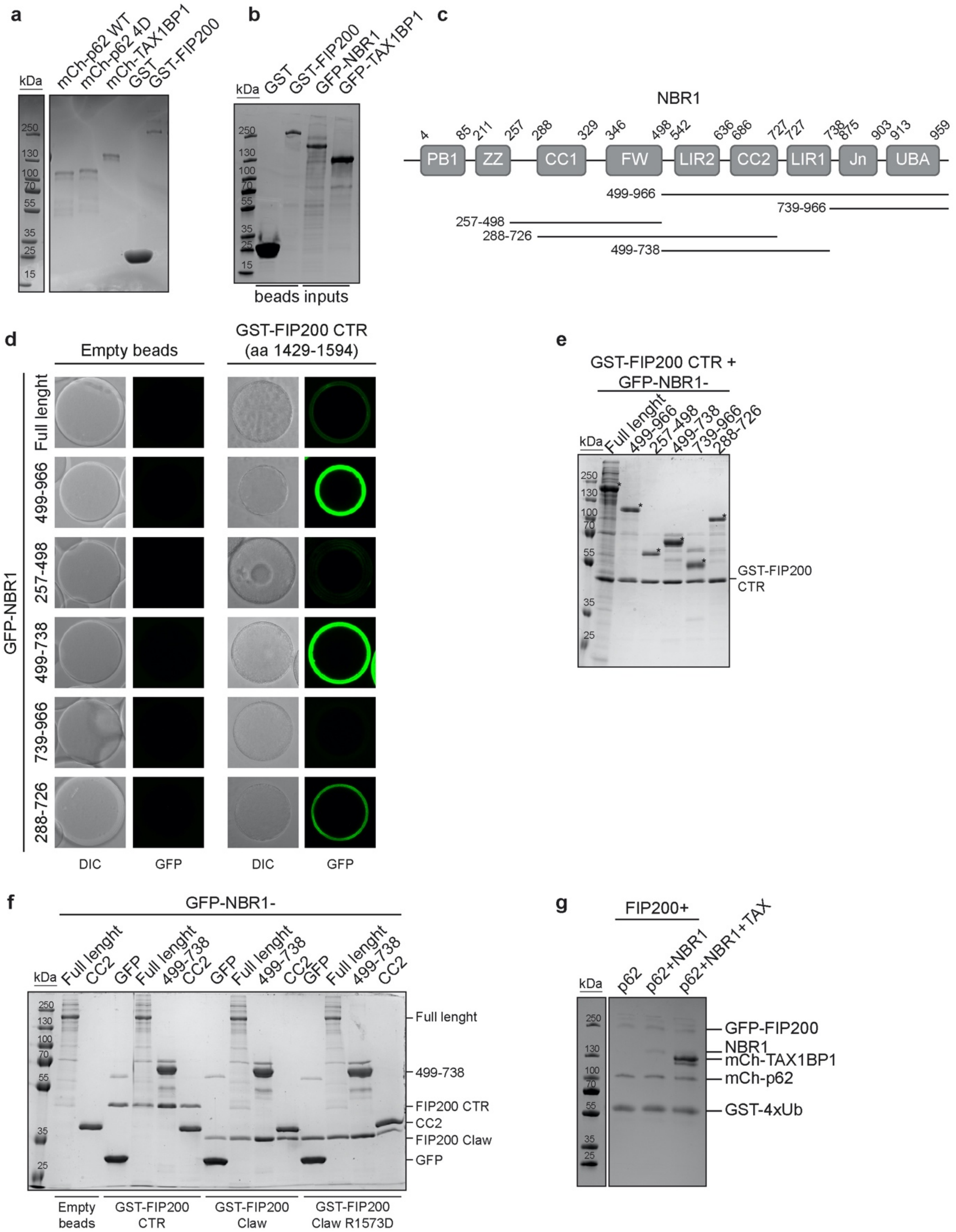

Supplementary figure 5.

a) 2 µM dilutions of the proteins used for the pull-down in Fig. 5a and 5 µg each of GST and GST-FIP200 were visualized by SDS-Page followed by Coomassie staining (n=3).

b) 2 μM dilutions of the proteins used for the pull-down in Fig. 5b and the GST/GST-FIP200 coupled beads (10 μl) were visualized by SDS-Page followed by Coomassie staining (n=3).
c) Schematic representation of NBR1 domains. The GFP-NBR1 constructs used for pull-downs in Supplementary fig. 5e and Fig. 5c are shown under the protein scheme.
d) Glutathione beads were left empty or coupled with GST-FIP200 CTR (aa 1429-1594) and incubated with the indicated GFP-NBR1 constructs (2 μM). Recruitment of GFP-NBR1 to the beads was visualized by confocal fluorescent microscopy (n=3). Protein inputs for the pull-down are shown in Supplementary fig. 5e.
e) Pull-down reactions for Supplementary fig. 5d were recovered from the microscopy plate and analyzed by SDS-Page followed by Coomassie staining (n=3). The * symbols indicate the protein band corresponding to each NBR1 construct.
f) Related to Fig. 5C. Pull-down reactions were recovered from the microscopy plate after imaging and analyzed by SDS-Page followed by Coomassie staining (n=3).
g) The condensates formation reactions from Fig. 5e were recovered from the microscopy plate after imaging and analyzed by SDS-Page followed by Coomassie staining (n=3).
Uncropped gels for panels a, b, e, f, g are provided as a Source Data file.

Supplementary figure 6

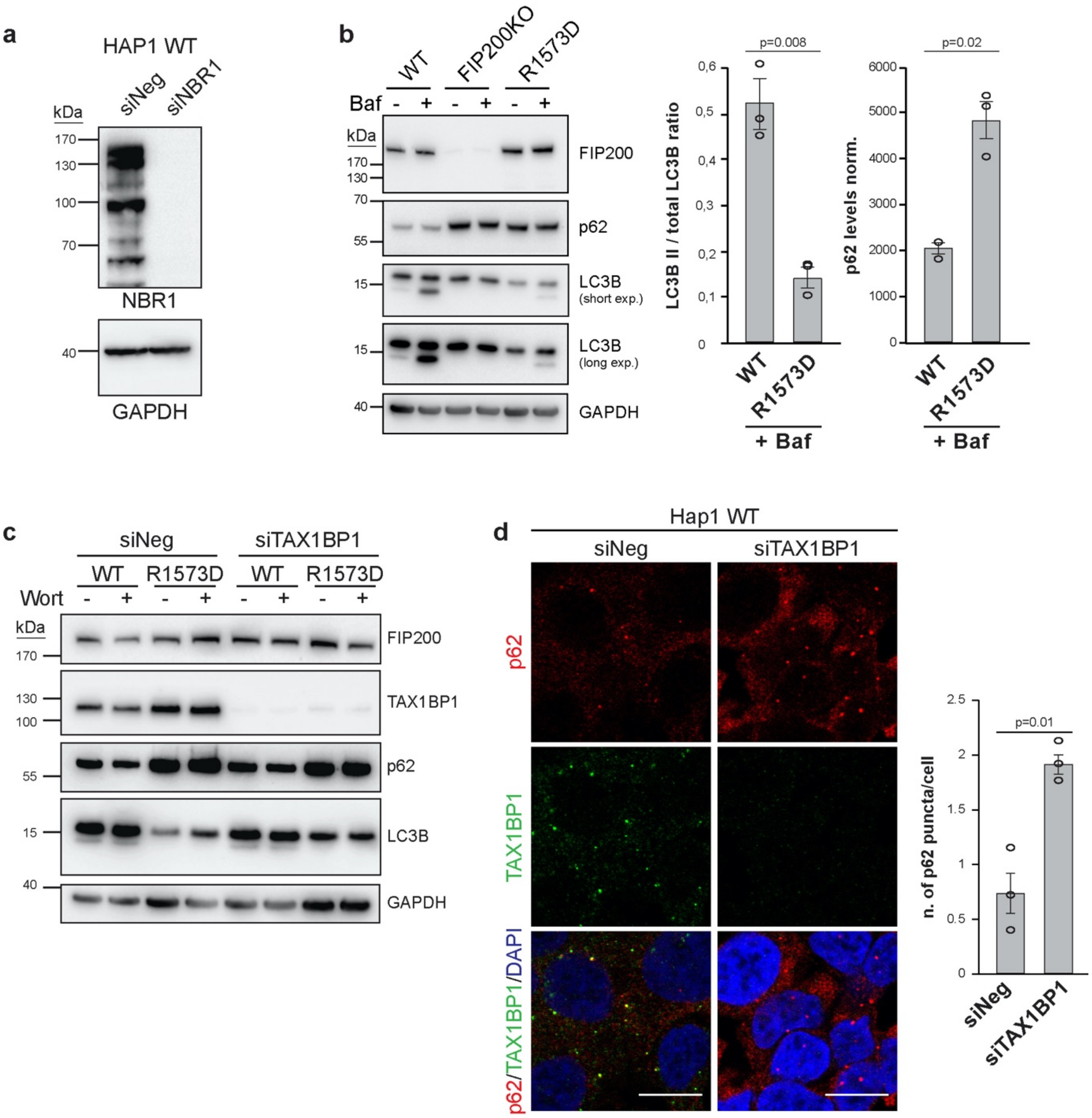

Supplementary figure 6.

a) Related to Fig. 6a. HAP1 WT cells were treated with non-targeting siRNA (siNeg) or with NBR1 siRNA (20 nM for 48 h). Cell lysates were analysed by western blot. The efficiency of the siRNA treatment was assessed with NBR1 antibody. GAPDH antibody was used as loading control (n=3).

b) Related to Fig. 6b. The R1573D mutation was introduced in endogenous FIP200 by CRISPR. HAP1 WT, FIP200ko and HAP1 FIP200 R1573D cells were left untreated (DMSO) or treated with bafilomycin (400 nM) for 2 h. Cell lysates were analyzed by western blot. The expression levels of FIP200 were assessed with FIP200 antibody and the levels of p62 and LC3B as autophagy markers were monitored with the respective antibodies. GAPDH antibody

was used as loading control. The intensity of the LC3B II and I bands was measured with Image J and the LC3BII/I ratio was plotted (left graph). The intensity of the p62 band was normalized to the intensity of GAPDH band and plotted (right graph). For both graphs, average band intensity/ratio and SEM for n = 3 are shown. An unpaired, two-tailed Student's t test was used to estimate significance. P values are indicated in the figure.

c) Related to Fig.6b. Cell lysates from HAP1 WT or FIP200 R1573D cells treated as described in Fig. 6b were analyzed by western blot. The efficiency of siRNA treatment was assessed with TAX1BP1 antibody. Other autophagy markers like p62 and LC3B, as well as FIP200 expression levels were monitored with the respective antibodies. GAPDH antibody was used as loading control (n=3).

d) Hap1 WT cells were treated with non-targeting siRNA (siNeg) or with TAX1BP1 siRNA (20 nM, 48h). Endogenous p62 and TAX1BP1 were detected by immunofluorescence staining. Scale bar = 10μM. The number of p62 puncta/cell was counted and plotted. Average p62 puncta number ± SEM for 3 independent experiments is shown. An unpaired, two-tailed Student's t test was used to estimate significance. P values are indicated in the figure.

Uncropped blots for panels a, b, c are provided as a Source Data file.

Supplementary Table 1: resource table.

REAGENT or RESOURCE	SOURCE	IDENTIFIER
**Antibodies**		
Mouse anti-p62	BD Bioscience	Cat#610832
Mouse monoclonal anti-NBR1	Abnova	Cat#H00004077-M01
Rabbit anti-FIP200 (D10D11)	Cell Signaling Technology	Cat#12436
Mouse anti-LC3B (clone 2G6)	nanoTools	Cat#0260-100
Mouse anti-GFP	Roche	Cat#11814460001
Mouse monoclonal anti-RFP (mScarlet)	Chromotek	Cat#6g6-100
Mouse anti-FLAG	Sigma	Cat#F3165-2MG
Rabbit monoclonal anti-TAX1BP1 (D1D59	Cell Signaling Technology	Cat#5105
Mouse anti-GAPDH	Sigma	Cat#G8795
Rabbit anti-p62	MBL	Cat#PM045
Mouse anti-Ubiquitin FK2	Enzo Life Science	Cat#BML-PW8810
Goat polyclonal anti-mouse HRP	Jackson Immunoresearch	Cat#115-035-003
Goat polyclonal anti-rabbit HRP	Jackson Immunoresearch	Cat#111-035-003
Goat anti-rabbit Alexa Fluor 488	Invitrogen	Cat#A11008
Goat anti-mouse Alexa Fluor 488	Invitrogen	Cat#A11001
Goat anti-mouse Alexa Fluor 647	Jackson Immunoresearch	Cat#115-605-146
Goat anti-rabbit Alexa Fluor 647	Jakcson Immunoresearch	Cat#111-605-144
**Bacterial and Virus Strains**		
E. coli Rosetta (DE3) pLys	Novagen	Cat#70956
E. coli DH10BacY	Gift from Leonard lab, Max Perutz lab, Vienna, Austria	-
**Chemicals, Peptides, and Recombinant Proteins**		
cOmplete EDTA-free protease inhibitor cocktail	Roche	Cat#11836170001
Bradford protein assay	Bio-Rad	Cat#5000006
Pierce BCA protein assay kit	ThermoFisher	Cat#23227
HisTrap 5 ml HP column	GE Healthcare	Cat#17524801
Glutathione Sepharose 4B beads	GE Healthcare	Cat#17075601
GFP-trap®_magnetic beads	Chromotek	Cat#gtma20
RFP-trap®_A beads	Chromotek	Cat#rta-20
Puromycin	ThermoFisher	Cat#A1113802
G-418 solution	Roche	Cat# 4727878001
MG132 (Z-Leu-Leu-Leu-CHO)	Boston Biochem	Cat#I-130
BafilomycinA1	Santa Cruz Biotech.	Cat#sc-201550
Wortmannin	Sigma	Cat#W1628
Lipofectamine® RNAiMAX Transfection Reagent	ThermoFisher	Cat#13778030
Fugene® 6 Transfection Reagent	Promega	Cat#E2691
DAPI-Fluoromount-G™	SouthernBiotech	Cat#0100-20
Protease Inhibitor Cocktail	Sigma	Cat#P8849
Pefabloc® SC-Protease inhibitor	Carl Roth	Cat#A154.3
Benzonase® Nuclease	Sigma-Aldrich	Cat#E1014-5KU

1-Naphthaleneacetic acid (1-NAA)	Sigma-Aldrich	Cat# N0640
LysoTracker Blue DND-22	Invitrogen	Cat#L7525
**Experimental Models: Cell Lines**		
HAP1 WT cells	Horizon Discovery	Cat#C631
HAP1 FIP200 KO cells	Horizon Discovery	Cat#HZGHC00056 7c007
HAP1 Strep-TEV-GFP-p62, mSc-AID-NBR1	This study	SMcl#65 (cl.B1)
HAP1 Strep-TEV-GFP-p62, mSc-AID-NBR1, TIR1-9myc	This study	SMcl#70 (cl.3D)
HAP1 Strep-TEV-GFP-p62, mSc-AID-NBR1, TIR1, 3xFLAG-iRFP-NBR1 WT	This study	SMcl#74 (cl.1)
HAP1 Strep-TEV-GFP-p62, mSc-AID-NBR1, TIR1, 3xFLAG-iRFP-NBR1 D50R	This study	SMcl#75 (cl.17)
HAP1 Strep-TEV-GFP-p62, mSc-AID-NBR1, TIR1, 3xFLAG-iRFP-NBR1 F929A	This study	SMcl#76 (cl.7)
HAP1 GFP-AID-NBR1	This study	SMcl#71 (cl.A1)
HAP1 FIP200-R1573D clone 7D	This study	SMcl83
**Experimental Models: Organisms/Strains**		
Spodoptera frugiperda (Sf9) cells	Gift from Leonard lab, Max Perutz Labs, Vienna, Austria	SF9 cells
**Oligonucleotides**		
Non-targeting siRNA pool ON-target Plus	Dharmacon	D-001810-10-50
NBR1 siRNA	Dharmacon	LQ-010522-00-0002
TAX1BP1 siRNA	Horizon discovery	LQ-016892-00-0002
FIP200-R1573D sgRNAF1: caccGACAGATTTAAAGTTCCTTTG	This study	SMP2199
FIP200-R1573D sgRNAR1: aaacCAAAGGAACTTTAAATCTGTC	This study	SMP2200
**Recombinant DNA**		
pGEX-GST-FIP200 CTR aa 1429-1594	[10]	SMC565
pGEX-GST-FIP200 Claw (aa 1494-1594)	This study	SMC1198
pGEX-GST-FIP200 Claw R1573D	This study	SMC1206
pET-His-TEV-mCherry-p62 WT	[26]	SMC391
pET-His-TEV-GFP-p62 WT	[20]	SMC390
pET-His-TEV-mCherry-p62-NBR1 chimera	This study	SMC1579
pFastBac-His-Strep-TEV-GFP-NBR1	[20]	SMC914
pFastBac-His-TEV-NBR1	This study	SMC912
pFastBac-His-Strep-TEV-GFP-NBR1ΔPB1	This study	SMC983
pFastBac-His-Strep-TEV-GFP-NBR1ΔUBA	This study	SMC985
pET-His-TEV-NBR1 PB1	This study	SMC550
pFastBac-His-GFP-NBR1 aa 499-966	This study	SMC1238
pET-His-TEV-GFP-NBR1 aa 257-498	This study	SMC1248
pET-His-TEV-GFP-NBR1 aa 499-738	This study	SMC1249
pET-His-TEV-GFP-NBR1 aa 739-966	This study	SMC1250
pET-His-TEV-GFP-NBR1 aa 288-726	This study	SMC1251
pET-His-TEV-GFP-NBR1 FW (aa 330-498)	This study	SMC1288
pET-His-TEV-GFP-NBR1-CC2 (aa 686-727)	This study	SMC1346

pLIB-GST-TAX1BP1	This study	SMC1434
pLIB-10xHis-TEV-mCherry-TAX1BP1	This study	SMC1436
pLIB-10xHis-TEV-GFP-TAX1BP1	This study	SMC1435
pGB-02-03-GST-FIP200	This study	SMC1446
pGB-02-03-GST-FIP200-GFP	This study	SMC1445
pGEX-4xUbiquitin	[26]	Gift from Ikeda lab (IMBA, Vienna, Austria)
pET-His-TEV-mCherry-p62 4P	[10]	SMC1035
pSpCas9n(BB)-2A-GFP (PX458)	Addgene plasmid	Cat#48138
pUC19	Addgene plasmid	Cat#50005
pETDuet-Uev1	Arrowsmith lab, Addgene	Cat#25619
pETDuet-SUMO-UbcH13	[37] Addgene	Cat#51131
pETDuet-UBA1	[20]	SMC915
pETDuet-CDC34A	[20]	SMC913
pETDuet-Ubiquitin	[20]	SMC907
AIO (containing sgRNAs for NBR1 endogenous tagging, Cas9D10A nickase, puromycin resistance)<br>Guide 1: GAGAGAAAAACACATTAGAA<br>Guide 2: GCCTCACAGCATGGAACCAC	This study	SMC1320
pUC19-NBR1homology-mScarlet-AID-NBR1 homology	This study	SMC1183
pUC19-NBR1homology-GFP-AID-NBR1 homology	This study	SMC1319
pBABE-TIR1-9Myc	[38], Addgene	Cat#47328
pInducer20-3xFLAG-iRFP-NBR1	This study	SMC1406
pInducer20-3xFLAG-iRFP-NBR1 PB1mut (D50R)	This study	SMC1407
pInducer20-3xFLAG-iRFP-NBR1 UBAmut (F929A)	This study	SMC1408
**Software and Algorithms**		
ImageJ 1.x	[36]	https://imagej.net/ImageJ1

Supplementary Table 2: primers list.

Primer name	Sequence	Purpose
pFastBac-His-TEV-NBR1		
SMP1733	ATATGTCGACAATGGAACCACAGGTTACTCTAAATG	Cloning of untagged NBR1 protein for purification
SMP1734	ATAT GCGGCCGC TCAATAGCGTTGGCTGTACC	Cloning of untagged NBR1 protein for purification
Generation of pFastBac-His-Strep-TEV-GFP-NBR1ΔPB1 and ΔUBA		
SMP1861	CACCATGTCGTTGATGAAGCCCCAC	Deletion of the PB1 domain from NBR1 for protein purification
SMP1862	TGGTTCCATGgtacCtcctccgc	Deletion of the PB1 domain from NBR1 for protein purification
SMP1865	AACAACGACTGGTACAGCCAACGCT	Deletion of the UBA domain from NBR1 for protein purification
SMP1866	AATTATTGGCTGTGCAGTGACTGGT	Deletion of the UBA domain from NBR1 for protein purification
NBR1 deletion constructs		
SMP2478	AAAACTGATGATCTCACCTGCCAGC	aa499-966
SMP2479	GgtacCtcctccgcttcctcctc	aa499-966
SMP2528	CCCCCCgcggccgcataatgcttaagtcgaa	pET-Duet-GFP
SMP2529	AAAAAAgtcgactgCTCGAGATCTGAGTCCGGA	pET-Duet-GFP
SMP2530	CCCCCCgtcgacAAGTTGCGGAGACCTGTTG	aa257-498
SMP2531	AAAAAAgcggccgcTCAGCTTGAGCTGATCATGCCC	aa257-498, FW
SMP2532	CCCCCCgtcgacAAAACTGATGATCTCACCTGCCAG	aa499-738
SMP2533	AAAAAAgcggccgcTCACTCAGGCAGGATGATGATG	aa499-738
SMP2534	CCCCCCgtcgacTGCTTTGATACCAGCCGCC	aa739-966
SMP2535	AAAAAAgcggccgcTCAATAGCGTTGGCTGTACCAG	aa739-966
SMP2536	CCCCCCgtcgacAGGCTCCAGAAACAGGTTGA	aa288-726
SMP2537	AAAAAAgcggccgcTCAAGAGGACTGACTTTGAACT	aa288-726
SMP2611	CCCCCCgtcgacCACCTGTGGAATTCAATCCATG	FW
SMP2712	CCCCCCgtcgacAATGAGAAGGAGGAGAT	CC2
SMP2713	AAAAAAgcggccgcTCAAGCAGAGGACTGACTTT	CC2
TAX1BP1 constructs		
SMP2916	ttgctgtcgacatgCATCACCATCATCATCACCATCATCACCACA GCCAGGATCCGAATTC	GFP/mCherry-TAX1BP1
SMP2915	GTGCTGAGCTCCTAGTCAAAATTTAGAACATTCTG	GFP/mCherry-TAX1BP1

SMP2918	ttgctgtcgacATGTCCCCTATACTAGG	GST-TAX1BP1
Hap 1 FIP200 R1573D mutation by CRISPR		
SMP2199	caccGACAGATTTAAAGTTCCTTTG	Guide 1
SMP2200	aaacCAAAGGAACTTTAAATCTGTC	Guide 2
SMP2585	ctattGGATCCTCTGGCAGTTATGTTTC	FIP200 genomic region
SMP2586	ataagCATATGCACACTTCCCAGCAATC	FIP200 genomic region
SMP2587	GACTTTAAAGTTCCTTTG	R1573D mutation
SMP2588	GTTTTGTGCCTAAGAG	R1573D mutation
Cloning of mSc-AID-NBR1 for CRISPR knock-in in Hap1 cells		
SMP2333	aaaagtcgacgttcattgttggggcagaagt	NBR1 genomic region
SMP2334	aaaaggatccgctgctacaattgttctgcaaag	NBR1 genomic region
SMP2322	caggagaatggcgtgaacccaggag	NBR1 genomic region
SMP2323	ctaatgtgtttttctctctctccca	NBR1 genomic region
SMP2324	ctggttatatctgatgataactaaa	NBR1 genomic region
SMP2325	gGGAGGCGGAGGAGGAGAACCACAGGTTACTCTAAATGTGACT	Gibson cloning of mScarlet-AID-NBR1
SMP2326	tcgcccttgctcacCATGCTGTGAGGctagggtt	Gibson cloning of mScarlet-AID-NBR1
SMP2327	CAGCATGgtgagcaagggcgagg	Gibson cloning of mScarlet-AID-NBR1
SMP2328	gtccgctagcCTTGTACAGCTCGTCCATGCCG	Gibson cloning of mScarlet-AID-NBR1
SMP2329	gctgtacaaggctagcggactcagatctcga	Gibson cloning of mScarlet-AID-NBR1
SMP2330	TTCtcctcctccgcctcccttc	Gibson cloning of mScarlet-AID-NBR1
SMP2331	gaccacgctgtaaatccctgg	mScarler-AID-NBR1 clone validation
SMP2332	agccataacatccagtgttgtca	mScarler-AID-NBR1 clone validation
SMP2365	ACC-G-AGAGAAAAACACATTAGAA	NBR1 guide 1
SMP2366	aaacTTCTAATGTGTTTTTCTCT	NBR1 guide 1
SMP2367	ACC-G-CCTCACAGCATGGAACCAC	NBR1 guide 2
SMP2368	aaacGTGGTTCCATGCTGTGAGG	NBR1 guide 2
SMP2436	TCCCACCAACCTTCTCAACC	NBR1 genomic region

SMP2437	TGGTTCCCTTTATTGGGGCA	NBR1 genomic region
SMP2467	TTTTTT-GGTACC-ATGGTGAGCAAGGGCGAGGC	Validation primers
SMP2468	CCCCCC-ttaattaa-TCAATAGCGTTGGCTGTACCAGTC	Validation primers
SMP2449	ATGGTGAGCAAGGGCGAGGC	Validation primers
SMP2450	TCAATAGCGTTGGCTGTACCAGTC	Validation primers
Cloning of pInducer20-3xFLAG-iRFP-NBR1 WT, D50R and F929A for stable dox-inducible re-expression in Hap1 cells		
SMP2841	CCCCC-GCGGCCGCAGTAGCAGGTCATGCCTCTGG	
SMP2842	AAAAA-GgtacCtcctccgcttcctcctccgcttcctccGCTCTCAAGCGCGGTGATCC	
SMP2843	CCCCC-GgtacCGAACCACAGGTTACTCTAAATGT	
SMP2844	AAAAA-ACCGGTTCAATAGCGTTGGCTGTACC	
SMP2847	gacgccTCAGAATTAACCATGGACTACAAAG	
SMP2848	tagactcgagTCAATAGCGTTGGCTGTACC	
SMP2849	AACGCTATTGActcgagtctagagggcccgc	
SMP2850	CATGGTTAATTCTGAggcgtctccaggcgatctga	
Screening for stable integration of pInducer20-3xFLAG-iRFP-NBR1 WT, D50R and F929A in Hap1 cells		
SMP2952	AGCTCGTTTAGTGAACCGTCA	
SMP2953	GGATGAAGCGCGGCAGATAA	
SMP2954	GGACAGCAGAGATCCAGTTTG	
SMP2955	TAGCGGTTGCCGAAATAGGA	
SMP2956	CGGGTTTATTACAGGGACAGCA	
SMP2957	ATACACCATCACCCGGTCGT	
SMP2908	GATCGGCAATCCCTCTACGG	
SMP2909	TGCCATCTTAAGCGCTTCTTC	
SMP2937	Cagggacagcagagatccag	
SMP2938	GCTGCCGCACGTACAGCT	
Sequencing primers		
SMP2291	GCTTCCTCAGAGGATTACATCATCATCCTGCCT	NBR1
SMP2711	GAGCTGCGGGCGATCTG	iRFP
SMP1790	TTGTGGGCTCCTCTGAACCG	NBR1
SMP1767	GATGATCTCACCTGCCAGCAAG	NBR1
SMP1766	ACTCTACTCCTCGTCTTCCT	NBR1
SMP1789	AGACTCTGGAAACAGTGCCC	NBR1
SMP2838	CGGCGAGGTGGATCTCTTCA	miRFP
SMP2910	GCTCTCAAGCGCGGTGATCC	iRFP
SMP2911	GAGAGGCGGTCCCGAATGCG	iRFP
SMP1770	CAGGAAACAGCTATGAC	M13 reverse primer for bacmid colony PCR screening
SMP1771	GTAAAACGACGGCCAG	M13 forward primer for bacmid colony PCR screening
