# Supplementary material for: Reconstitution defines the roles of p62, NBR1 and TAX1BP1 in ubiquitin condensate formation and autophagy initiation
Source: Nat Commun. 2021 Sep 1;12:5212. doi: 10.1038/s41467-021-25572-w (PMC8410870; doi:10.1038/s41467-021-25572-w)
Supplement: Supplementary file 4 — Description of additional supplementary files [file 41467_2021_25572_MOESM4_ESM.docx]

Description of additional supplementary items

Title: Supplementary video 1

Description: The HAP1 GFP-p62 mScarlet-AIDNBR1 cell line was imaged under basal conditions (only in IMDM media), at a temperature of 37oC temperature and at 5% CO2. One image per Z plane was taken approx. every 1 second over a period of one minute. The GFP signal was thresholded such that diffuse p62 is visible in the cytosol of the cells and p62 puncta are seen as mobile condensates
