# Supplementary material for: Reconstitution defines the roles of p62, NBR1 and TAX1BP1 in ubiquitin condensate formation and autophagy initiation
Source: Nat Commun. 2021 Sep 1;12:5212. doi: 10.1038/s41467-021-25572-w (PMC8410870; doi:10.1038/s41467-021-25572-w)
Supplement: Supplementary file 5 — Reporting Summary [file 41467_2021_25572_MOESM5_ESM.pdf]

nature research

Corresponding author(s): Sascha Martens, Eleonora Turco

Last updated by author(s): Jul 22, 2021

Reporting Summary

Nature Research wishes to improve the reproducibility of the work that we publish. This form provides structure for consistency and transparency in reporting. For further information on Nature Research policies, see our Editorial Policies and the Editorial Policy Checklist.

Statistics

For all statistical analyses, confirm that the following items are present in the figure legend, table legend, main text, or Methods section.

n/a	Confirmed	
☐	☒	The exact sample size (n) for each experimental group/condition, given as a discrete number and unit of measurement
☐	☒	A statement on whether measurements were taken from distinct samples or whether the same sample was measured repeatedly
☐	☒	The statistical test(s) used AND whether they are one- or two-sided<br>*Only common tests should be described solely by name; describe more complex techniques in the Methods section.*
☐	☒	A description of all covariates tested
☐	☒	A description of any assumptions or corrections, such as tests of normality and adjustment for multiple comparisons
☐	☒	A full description of the statistical parameters including central tendency (e.g. means) or other basic estimates (e.g. regression coefficient) AND variation (e.g. standard deviation) or associated estimates of uncertainty (e.g. confidence intervals)
☐	☒	For null hypothesis testing, the test statistic (e.g. F, t, r) with confidence intervals, effect sizes, degrees of freedom and P value noted<br>*Give P values as exact values whenever suitable.*
☒	☐	For Bayesian analysis, information on the choice of priors and Markov chain Monte Carlo settings
☒	☐	For hierarchical and complex designs, identification of the appropriate level for tests and full reporting of outcomes
☒	☐	Estimates of effect sizes (e.g. Cohen's d, Pearson's r), indicating how they were calculated

Our web collection on statistics for biologists contains articles on many of the points above.

Software and code

Policy information about availability of computer code

Data collection	For data extraction from the condensate formation assay a custom ImageJ macro was used. The macro is available in Zaffagnini et al., 2018 (doi: 10.15252/embj.201798308) as "code EV1".
Data analysis	Data analysis was performed with Fiji/ImageJ v 1.0, available as open source at www.imagej.net

For manuscripts utilizing custom algorithms or software that are central to the research but not yet described in published literature, software must be made available to editors and reviewers. We strongly encourage code deposition in a community repository (e.g. GitHub). See the Nature Research guidelines for submitting code & software for further information.

Data

Policy information about availability of data

All manuscripts must include a data availability statement. This statement should provide the following information, where applicable:

- Accession codes, unique identifiers, or web links for publicly available datasets
- A list of figures that have associated raw data
- A description of any restrictions on data availability

The authors declare that the data supporting the findings of this study are available within the paper and its supplementary information files. Source data are included with this paper. The source data file includes all the datasets for the condensate formation assay and all the uncropped gels and western blots.

April 2020

Field-specific reporting

Please select the one below that is the best fit for your research. If you are not sure, read the appropriate sections before making your selection.

☒ Life sciences ☐ Behavioural & social sciences ☐ Ecological, evolutionary & environmental sciences

For a reference copy of the document with all sections, see nature.com/documents/nr-reporting-summary-flat.pdf

Life sciences study design

All studies must disclose on these points even when the disclosure is negative.

Sample size	For the quantification of immunofluorescence experiments between 60 and 100 cells per sample/condition were used for analysis in order to represent the cell population. For the microscopy based pull down assays between 50 and 80 beads/conditions were analyzed. In all cases 3 independent experiments were performed and the avarage value between the replicates was given as final result.
Data exclusions	No data were excluded from the analysis
Replication	To ensure reproducibility, the experiments showed in the manuscript were repeated at least 3 times with all attempts of replication being successful
Randomization	Randomization is not applicable for cell lines with different treatments or protein samples.
Blinding	Investigators were not blinded as the readouts were not subjective. The same scientist designed the experiments, executed them and performed the data analysis.

Reporting for specific materials, systems and methods

We require information from authors about some types of materials, experimental systems and methods used in many studies. Here, indicate whether each material, system or method listed is relevant to your study. If you are not sure if a list item applies to your research, read the appropriate section before selecting a response.

Materials & experimental systems

n/a	Involved in the study	
☐	☒	Antibodies
☐	☒	Eukaryotic cell lines
☒	☐	Palaeontology and archaeology
☒	☐	Animals and other organisms
☒	☐	Human research participants
☒	☐	Clinical data
☒	☐	Dual use research of concern

Methods

n/a	Involved in the study	
☒	☐	ChIP-seq
☒	☐	Flow cytometry
☒	☐	MRI-based neuroimaging

Antibodies

Antibodies used

Primary antibodies:
Mouse anti-p62, BD Bioscience - Cat# 610832
Mouse Monoclonal anti-NBR1 (6B11), Abnova - Cat#H00004077M01
Rabbit anti-FIP200 (D10D11), Cell Signaling Technology - Cat#12436
Mouse anti-LC3B, nanoTools - Cat#0260-100
Mouse anti-GFP, Roche - Cat#11814460001
Mouse monoclonal anti-RFP (mScarlet)cl.6G6, Chromotek - Cat#6g6-100
Mouse anti-FLAG, Sigma - Cat#F3165-2MG
Rabbit Monoclonal anti-TAX1BP1 (D1D59), Cell signaling Technology - Cat#5105
Mouse anti-GAPDH, Sigma - Cat#G8795
Rabbit anti-p62, MBL - Cat#PM045
Mouse anti-Ubiquitin FK2, Enzo Life Science - Cat#ENZ-ABS840

Secondary antibody:
Goat polyclonal anti-mouse HRP, Jackson Immunoresearch - Cat#115-035-003
Goat polyclonal anti-rabbit HRP, Jackson Immunoresearch - Cat#111-035-003
Goat anti-rabbit AlexaFluor 488, Invitrogen - Cat#A11008
Goat anti-mouse AlexaFluor 488, Invitrogen - Cat#A11001
Goat anti-mouse AlexaFluor 647, Jackson Immunoresearch - Cat#115-605-146
Goat anti-rabbit AlexaFluor 647, Jackson Immunoresearch - Cat#111-605-144

Validation	- Mouse anti-p62 (BD Bioscience): tested by the manufactured for WB and IF (https://www.bdbiosciences.com/us/reagents/research/antibodies-buffers/cell-biology-reagents/cell-biology-antibodies/purified-mouse-anti-p62-ick-ligand-3p62-lck-ligand/p/610832). Further validated (p62 detection in WT and p62 knockdown cells) in Wurzer et al., 2015 (doi: 10.7554/eLife.08941).<br>- Mouse monoclonal anti-NBR1 (Abnova): for validation see figure S1 of this study where the antibody was used to detect endogenous NBR1 (WT or fused to a tag). In Figure S6 the antibody was also used to detect NBR1 in WT and siRNA treated HAP1 cells.<br>- Rabbit anti FIP200 (Cell Signaling): the antibody was characterized by the manufacturer (https://www.cellsignal.at/products/primary-antibodies/fip200-d10d11-rabbit-mab/12436). Further validation for the use in IF was done in Turco et al., 2019 (doi: 10.1016/j.molcel.2019.01.035). The antibody was also used to detect endogenous FIP200 by WB in HAP1 WT and FIP200 KO cells (this study - Figure S6).<br>- Mouse anti-LC3B (nano-Tools): the antibody was validated by the manufacturer using positive controls (http://www.nanotools.de/shop/artikel/p209_0231-100_LC3-5F10.php). This antibody is used routinely in the laboratory for the detection of LC3B by WB and IF (see also Fig. 1 and Supplementary Fig. 6 of this study).<br>- Rabbit anti TAX1BP1 (Cell Signaling): validation for WB was performed by detecting TAX1BP1 in HAP1 cells WT and treated with TAX1BP1 siRNA (this study, supplementary fig. 6). The same experiment was performed in HeLa cells (data not shown). For detection of TAX1BP1 by IF, HAP1 cells WT and treated with TAX1BP1 siRNA were used. No TAX1BP1 puncta were detected in the siRNA treated sample (data not shown).<br>- Rabbit anti-p62 (MBL): the antibody is extensively used for the detection of p62 by IF and WB as shown in the references indicated in the product page (https://www.mblbio.com/bio/g/dtl/A/?pcd=PM045#u-pub).<br>- Mouse anti-ubiquitin FK2: the antibody was validated by the manfactured (https://www.enzolifesciences.com/fileadmin/reports/Datasheet-ENZ-ABS840.pdf) and it is extensively used in the ubiquitin field for the detection of ubiquitin conjugates.<br>Antibodies against tags were used in this study to detect endogenously tagged protein where the presence of the tag was previously confirmed by sequencing.

Eukaryotic cell lines

Policy information about cell lines

Cell line source(s)	The following cell lines were purchased from Horizon Discovery (https://horizondiscovery.com/en/engineered-cell-lines/products/human-hap1-knockout-cell-lines):<br>HAP1 WT - Cat#C631<br>HAP1 FIP200 KO - Cat#HZGHC000567c007<br>The following cell lines were generated in this study:<br>HAP1 Strep-TEV-GFP-p62, mSc-AID-NBR1 cloneB1 (SMcl#65)<br>HAP1 Strep-TEV-GFP-p62, mSc-AID-NBR1, TIR1-9xmyc clone 3D (SMcl#70)<br>HAP1 Strep-TEV-GFP-p62, mSc-AID-NBR1, TIR1, 3xFLAG-iRFP-NBR1 WT clone 1 (SMcl#74)<br>HAP1 Strep-TEV-GFP-p62, mSc-AID-NBR1, TIR1, 3xFLAG-iRFP-NBR1 F929A clone 7 (SMcl#76)<br>HAP1 Strep-TEV-GFP-p62, mSc-AID-NBR1, TIR1, 3xFLAG-iRFP-NBR1 D50R clone 17 (SMcl#75)<br>HAP1 GFP-AID-NBR1 clone A1 (SMcl71)<br>HAP1 FIP200 R1573D clone 7D (SMcl83)
Authentication	The cell lines was not authenticated because all cell lines generates for this study used were derived from the HAP1 WT cell lines directly purchased from Horizon Discovery as parental cell line.
Mycoplasma contamination	All cell lines used in the study were regularly checked for Mycoplasma contamination and they tested negative.
Commonly misidentified lines<br>(See ICLAC register)	No commonly misidentified lines were used in this study.
