# Supplementary material for: Reconstitution defines the roles of p62, NBR1 and TAX1BP1 in ubiquitin condensate formation and autophagy initiation
Source: Nat Commun. 2021 Sep 1;12:5212. doi: 10.1038/s41467-021-25572-w (PMC8410870; doi:10.1038/s41467-021-25572-w)
Supplement: Supplementary file 6 — Source data [file 41467_2021_25572_MOESM6_ESM.zip › Source data/Source data_Uncropped gels and blots.pdf]

Figure 5D

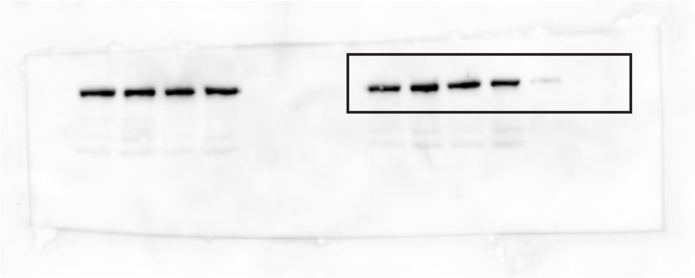

FIP200 (short exp.)

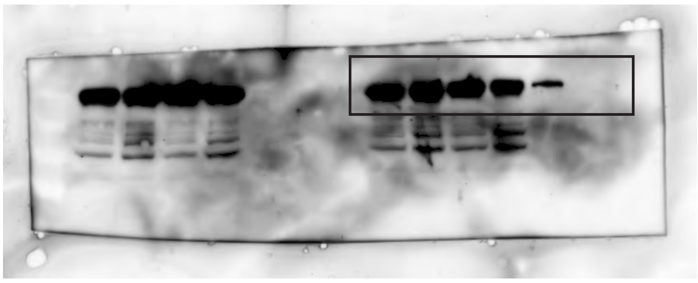

FIP200 (long exp.)

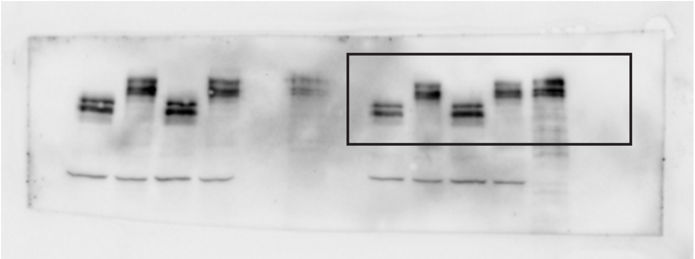

NBR1

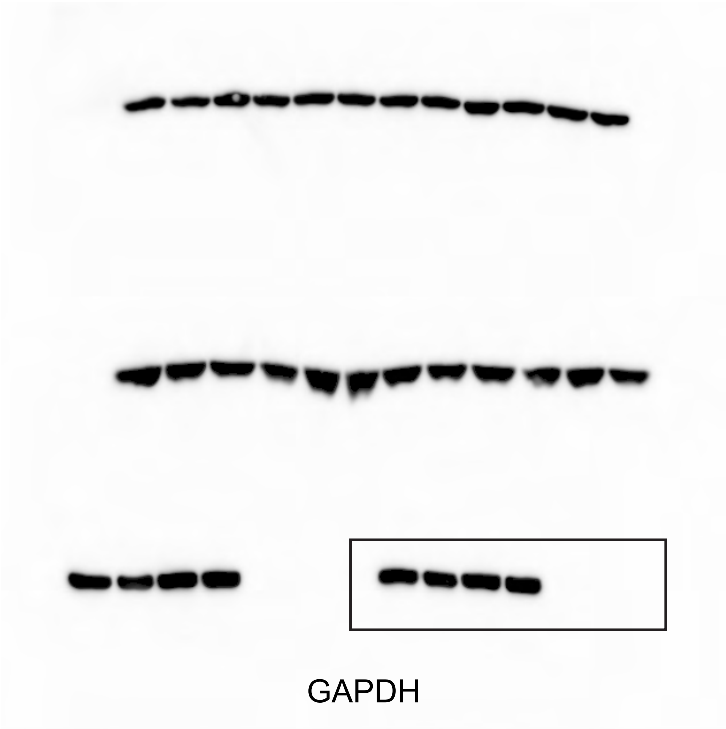

GAPDH

Supplementary figure 1C

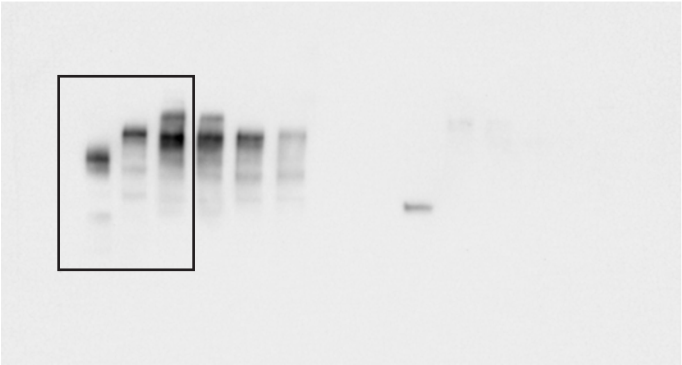

NBR1

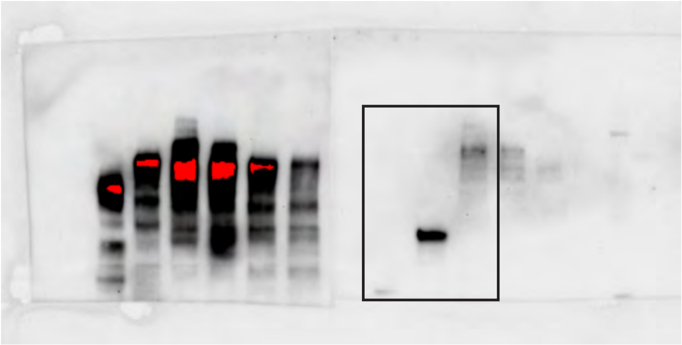

GFP

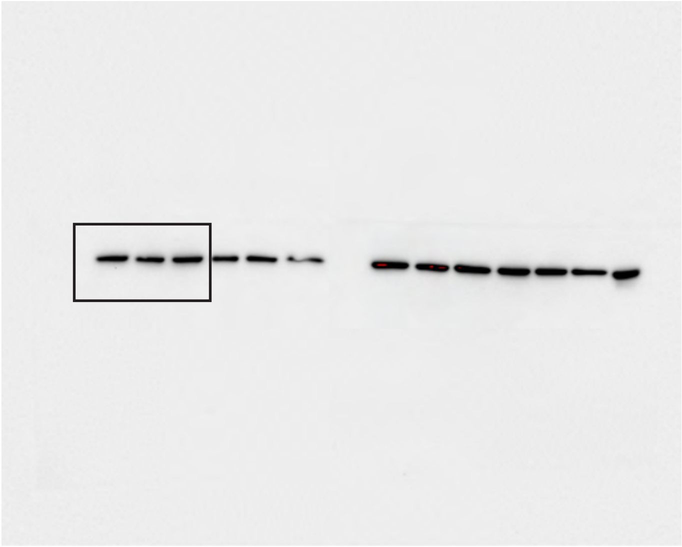

GAPDH

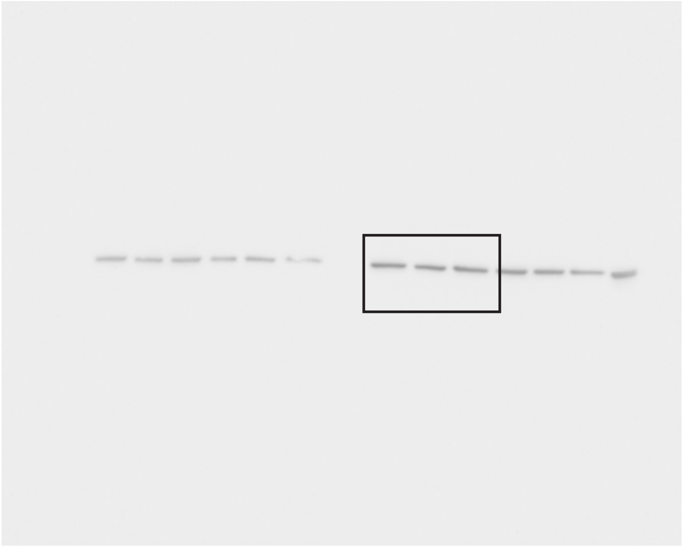

GAPDH

Supplementary figure 1D

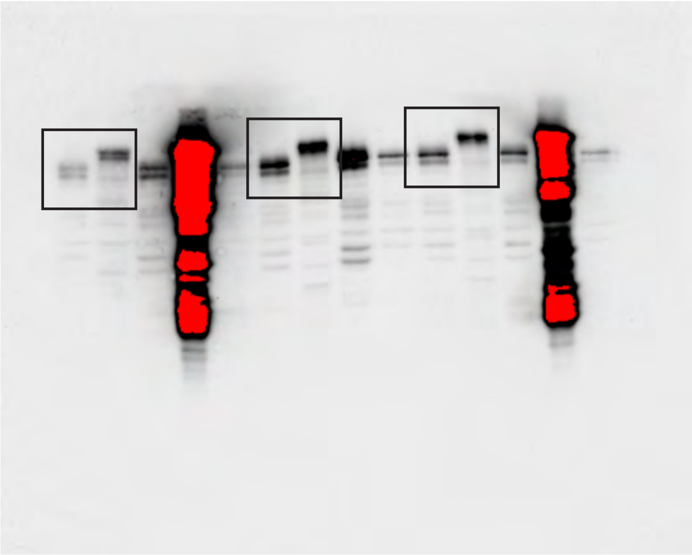

NBR1

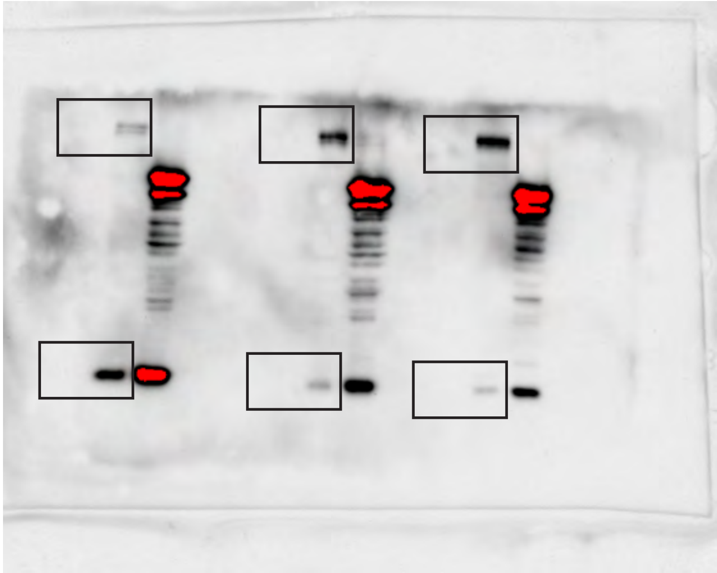

mScarlet (150kDa-30kDa)

Supplementary figure 2B

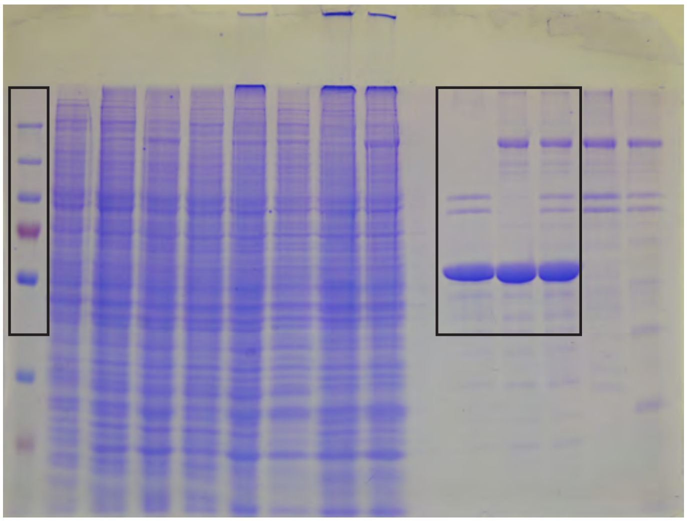

Supplementary figure 2C

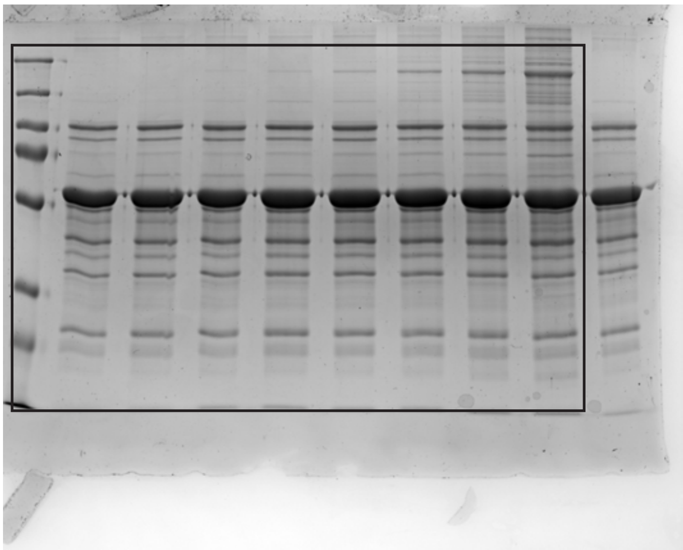

Supplementary figure 2D

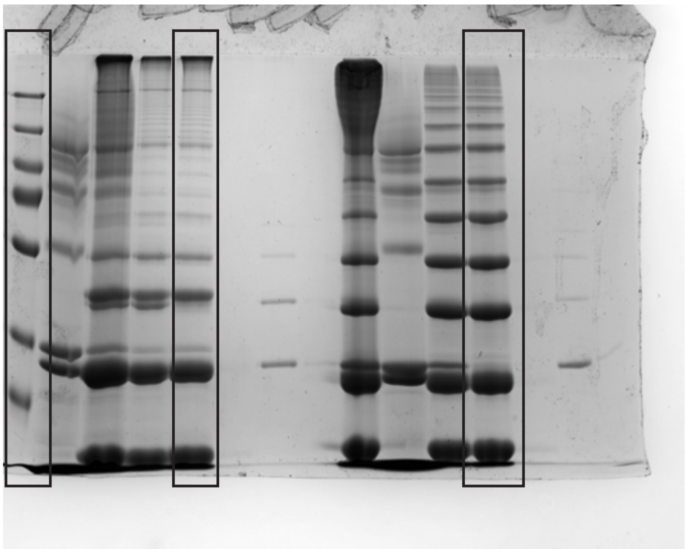

Supplementary figure 2E

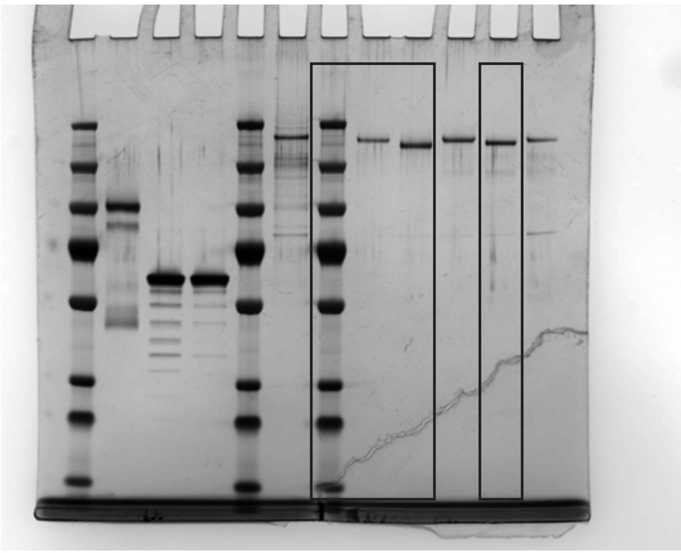

Supplementary figure 2F

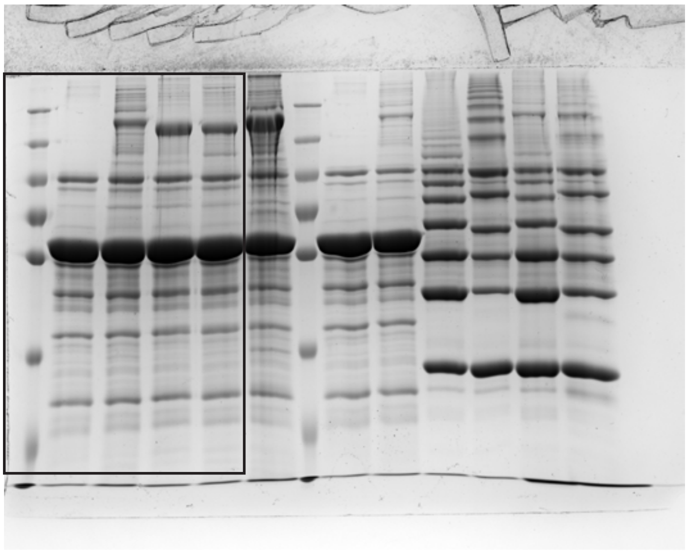

Supplementary figure 2H

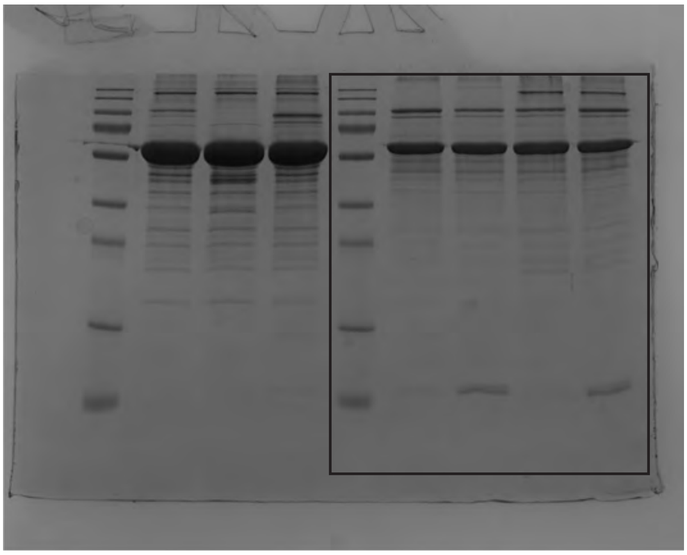

Supplementary figure 3A

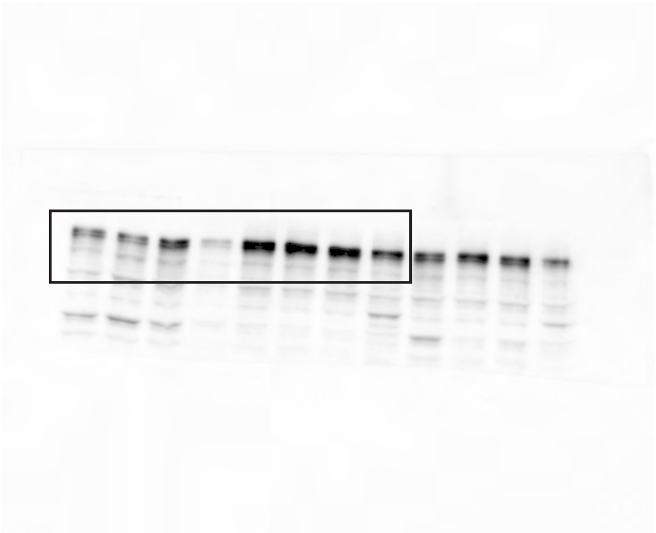

NBR1

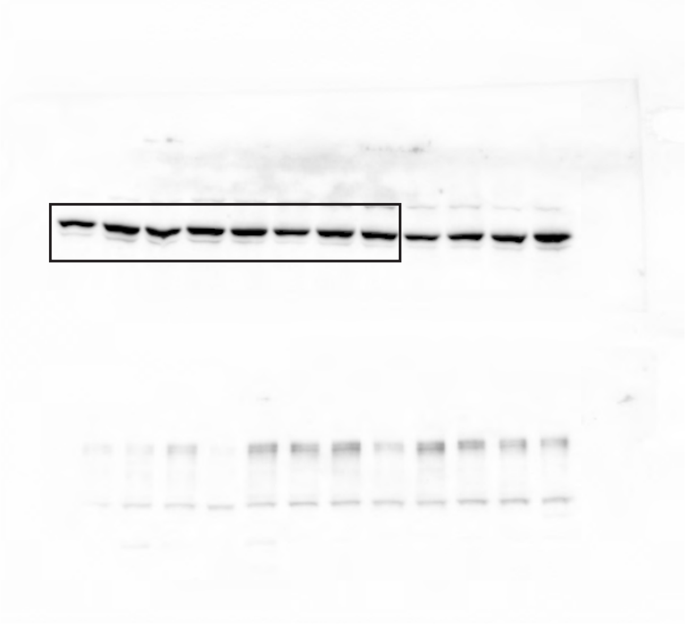

p62

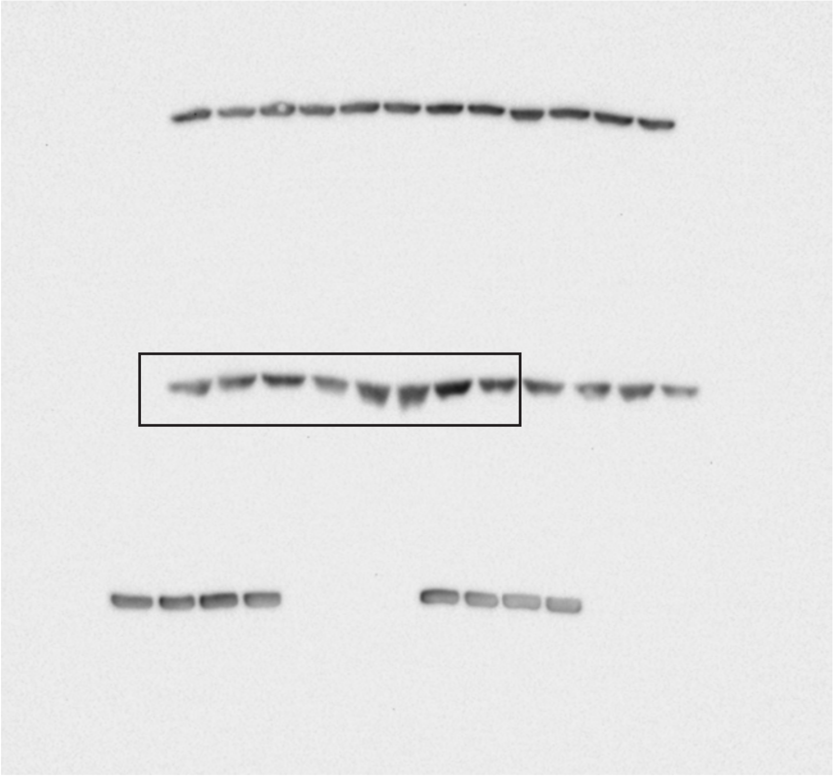

GAPDH

Supplementary figure 3C

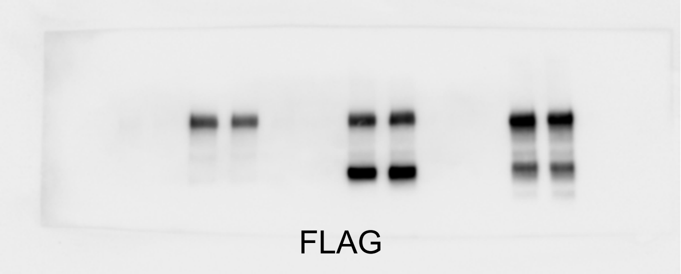

FLAG

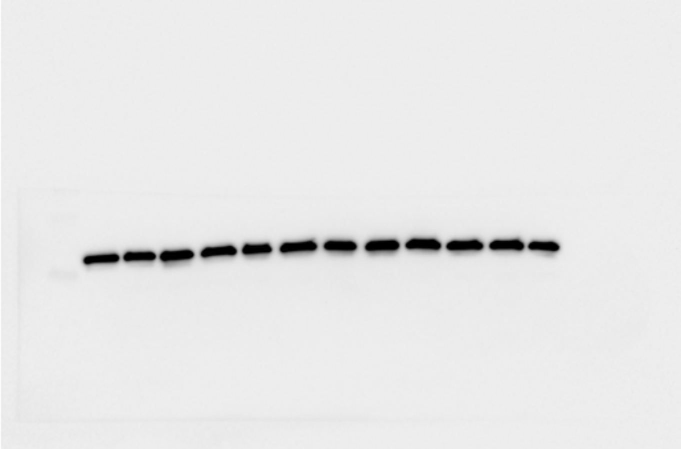

GAPDH

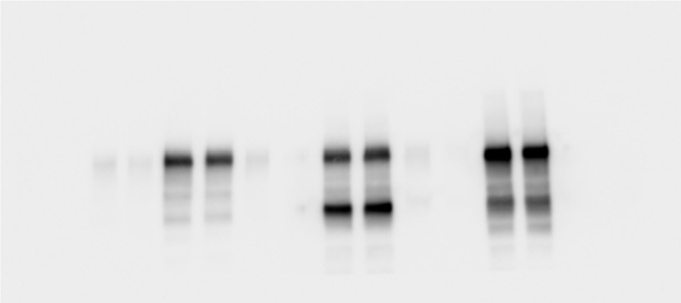

NBR1 (short exp.)

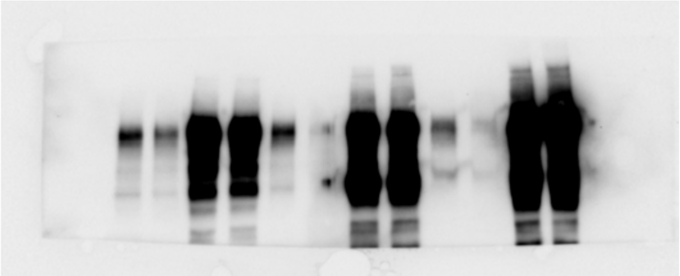

NBR1 (long exp.)

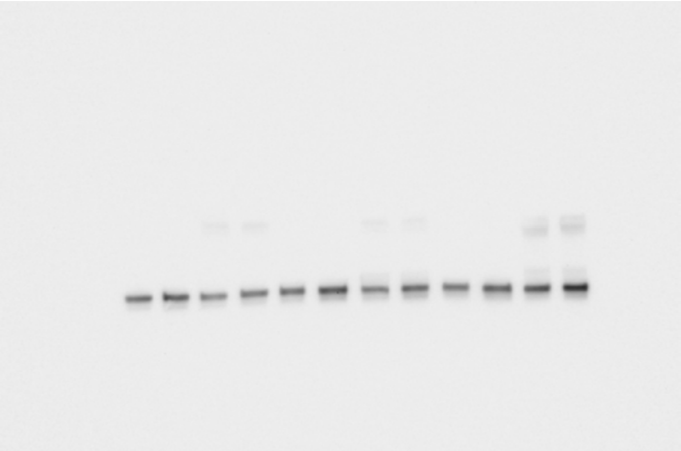

p62

Supplementary figure 4B

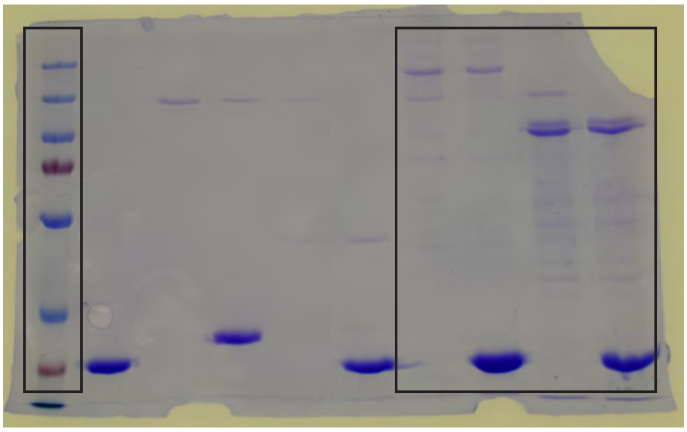

Supplementary figure 4E

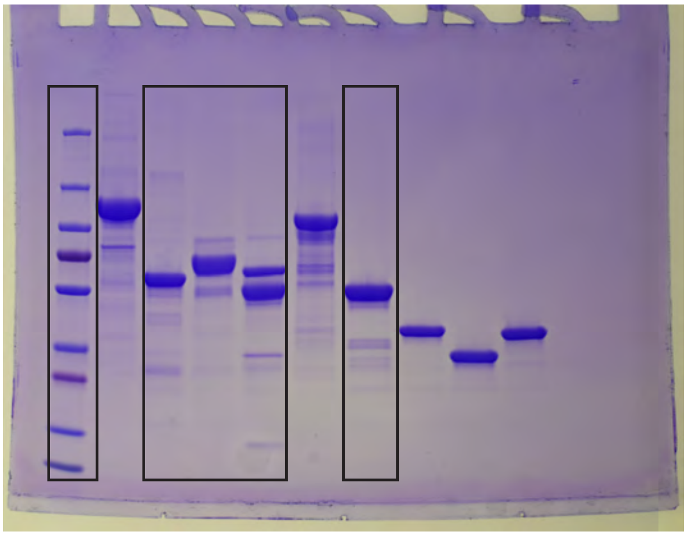

Supplementary figure 4G

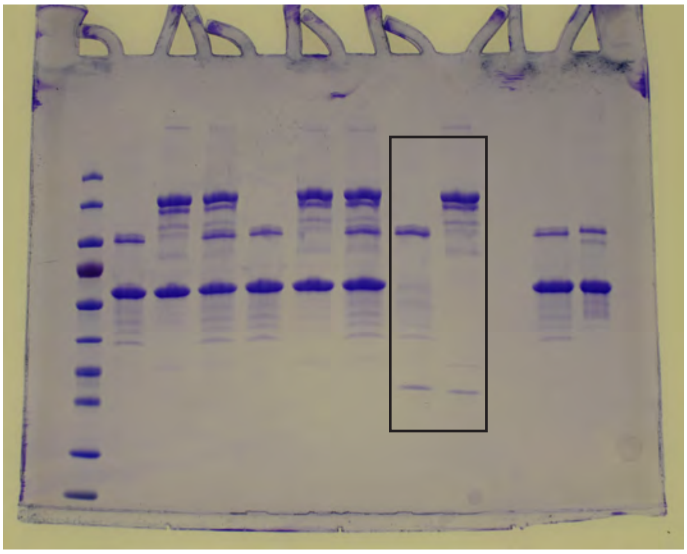

Controls

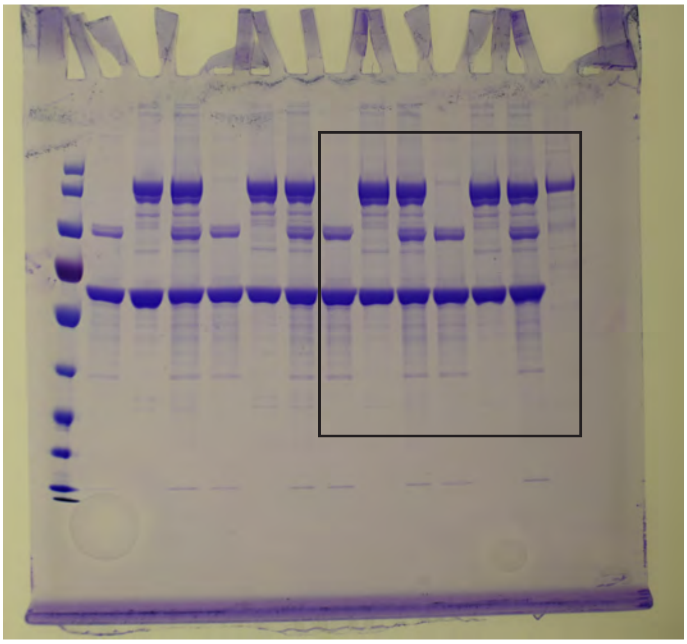

Reactions

Supplementary figure 4I

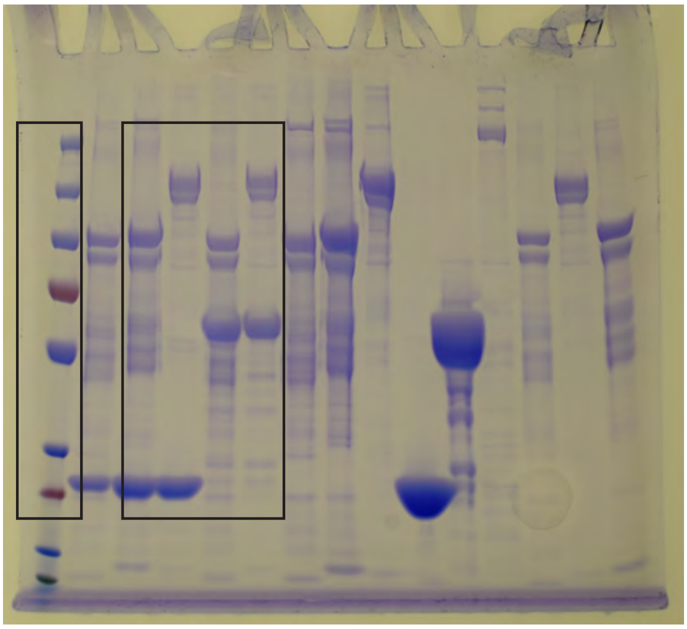

Supplementary figure 5A

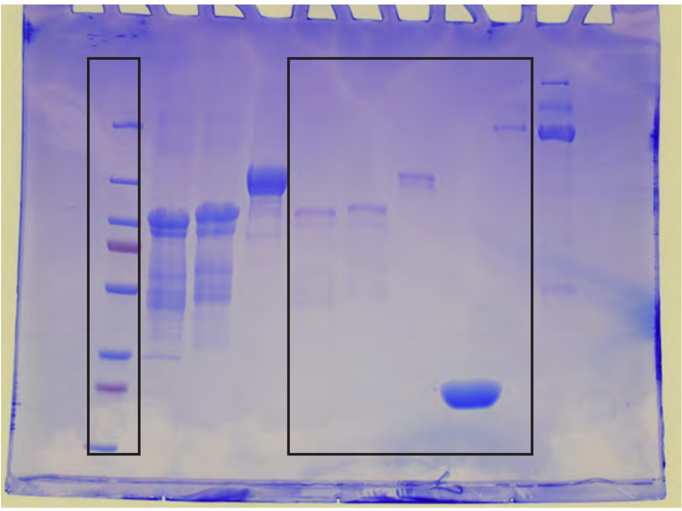

Supplementary figure 5B

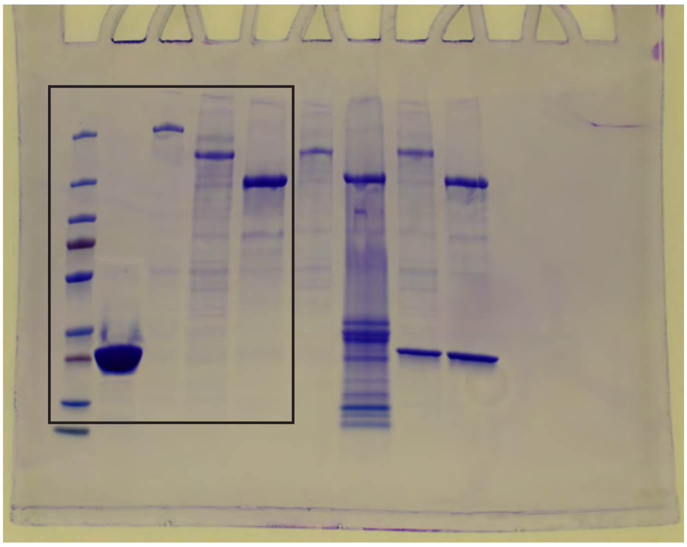

Supplementary figure 5E

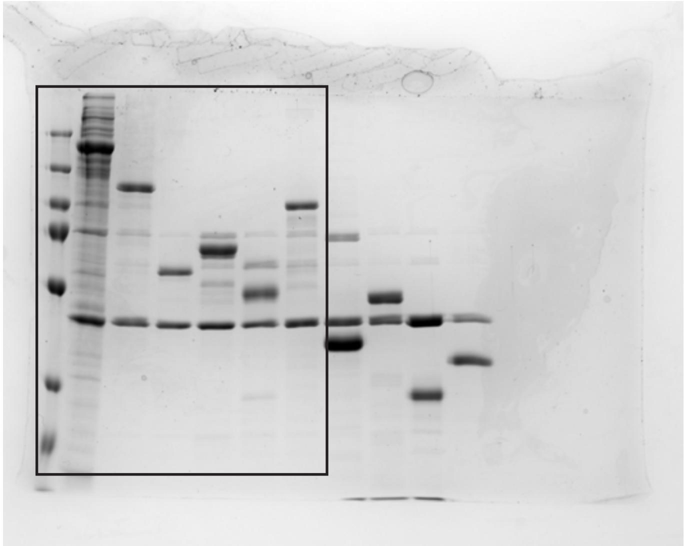

Supplementary figure 5F

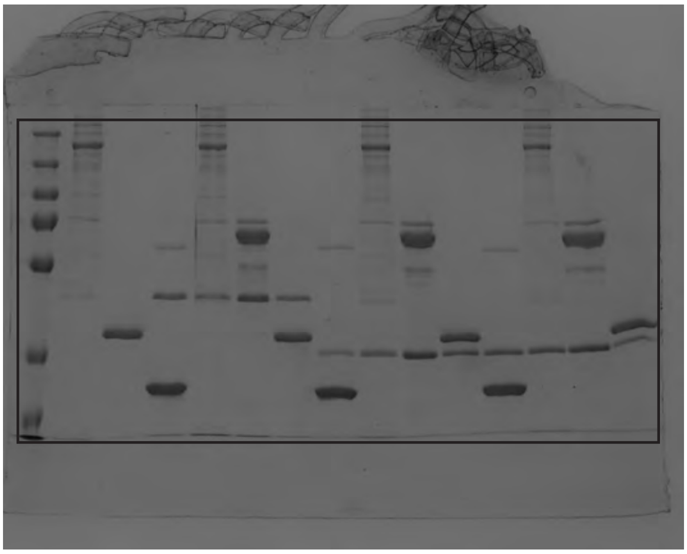

Supplementary figure 5G

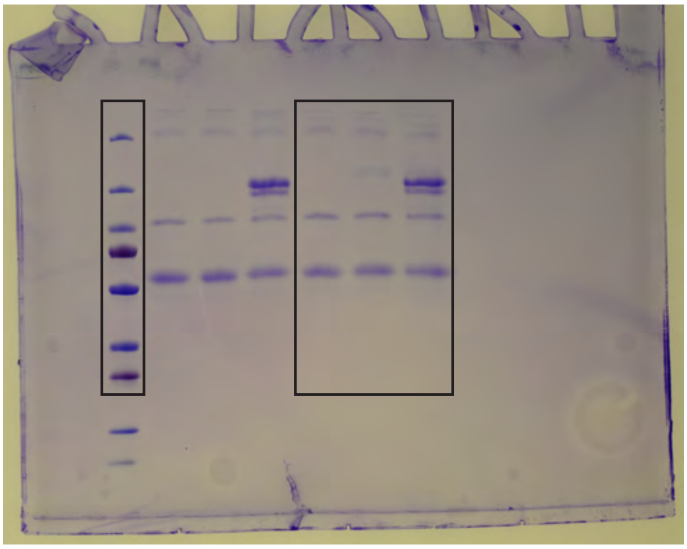

Supplementary figure 6A

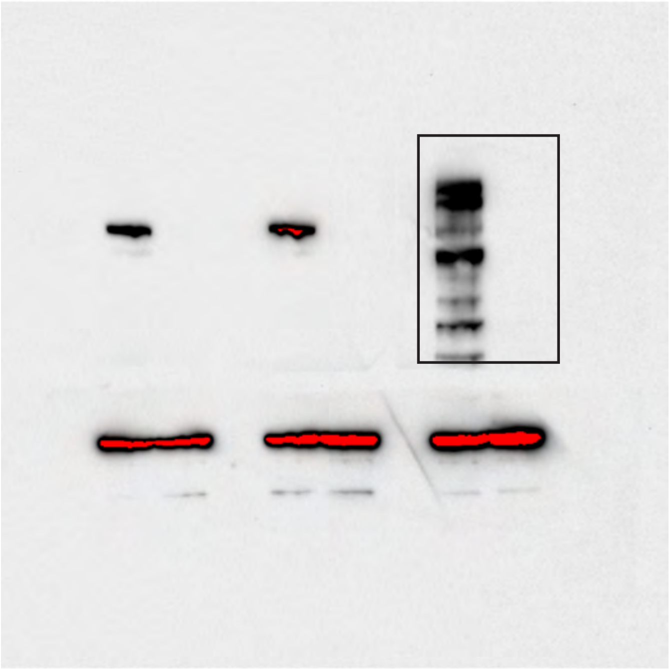

NBR1

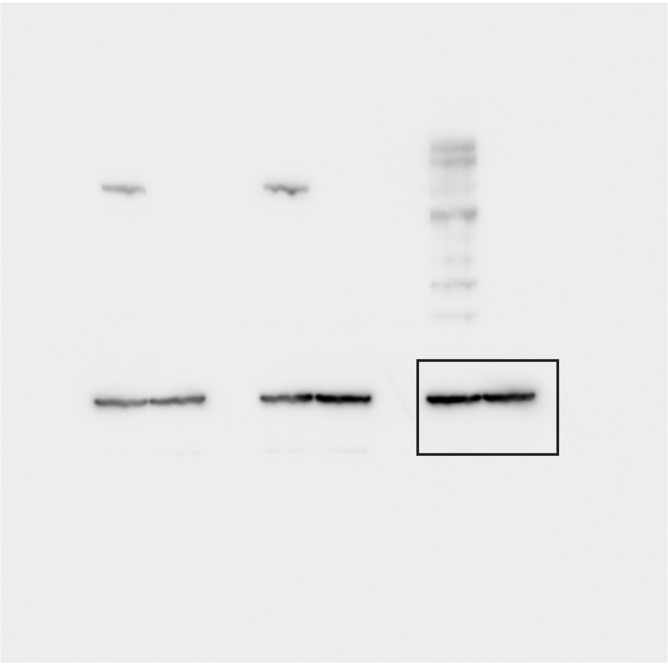

GAPDH

Supplementary figure 6B

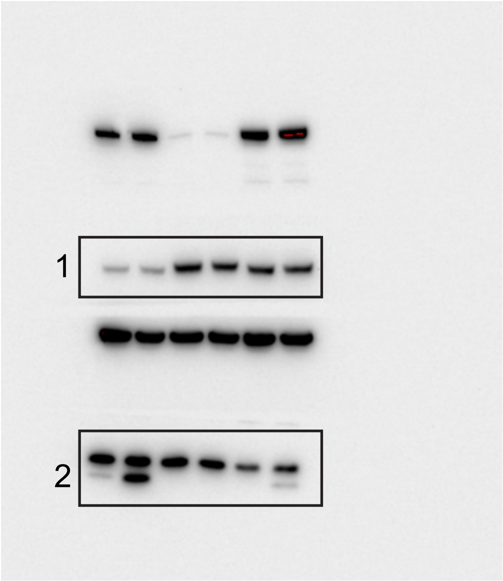

1-p62, 2-LC3B (long exp.)

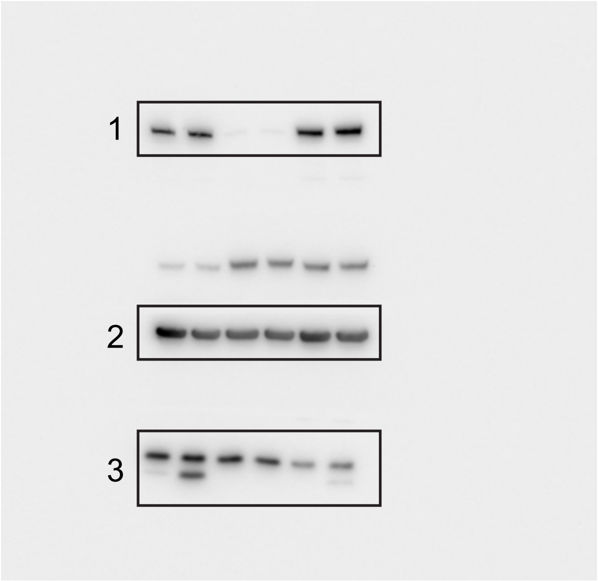

1-FIP200, 2-GAPDH, 3-LC3B (short exp.)

Supplementary figure 6C

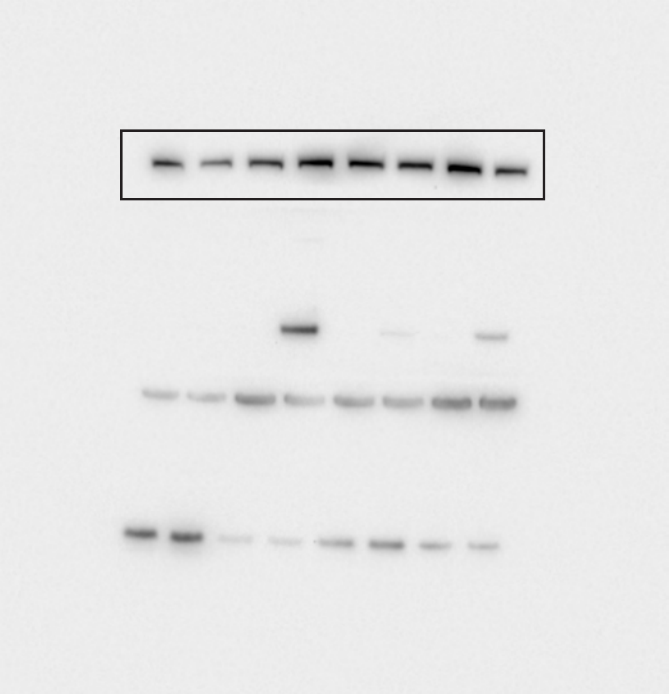

FIP200

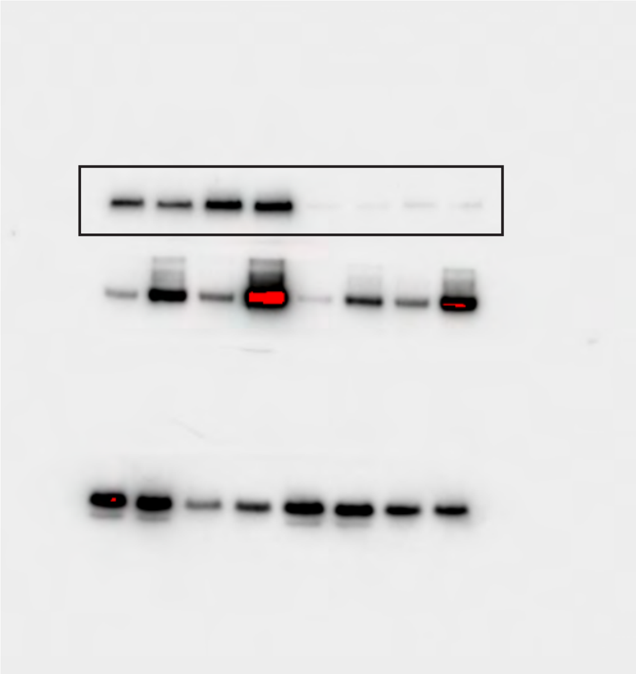

TAX1BP1

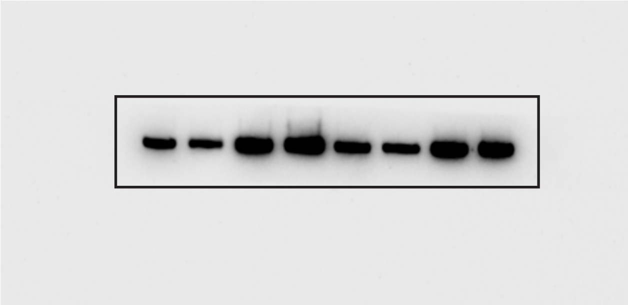

p62

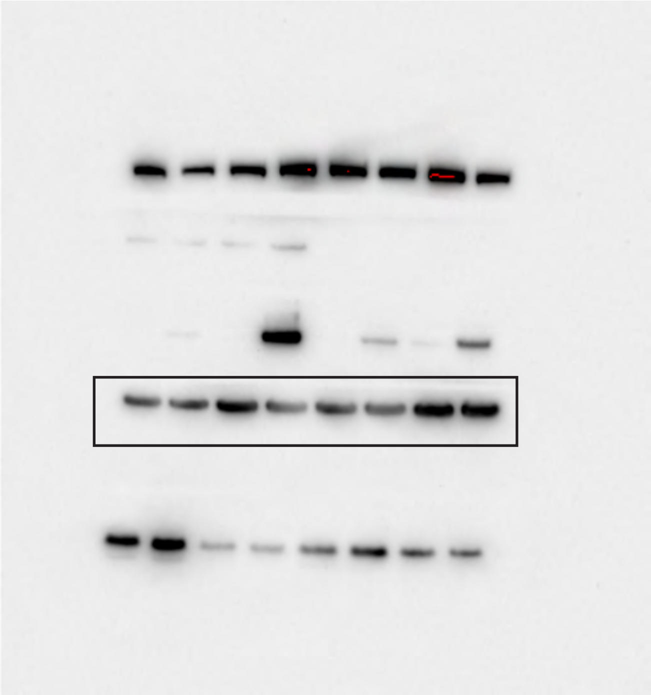

GAPDH

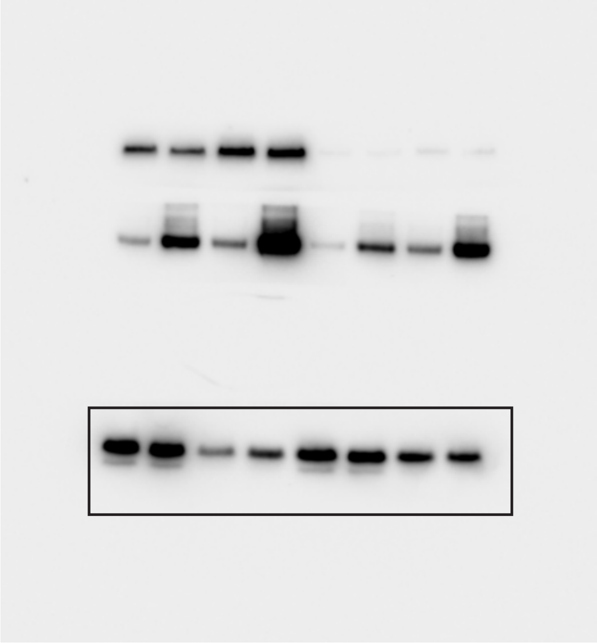

LC3B
